# Rosemary-derived triterpene acids improve growth and lipid metabolism in juvenile grass carp (*Ctenopharyngodon idella*) through the gut–liver axis by tissue-specifically regulating the farnesoid X receptor

**DOI:** 10.1186/s40104-025-01351-1

**Published:** 2026-03-12

**Authors:** Zixuan Wu, Xiaoqiu Zhou, Lin Feng, Pei Wu, Hongyun Zhang, Yaobin Ma, Yang Liu, Caimei Wu, Jiayong Tang, Ruinan Zhang, Weidan Jiang

**Affiliations:** 1 Animal Nutrition Institute, Sichuan Agricultural University, Chengdu, 611130 China; 2 Fish Nutrition and Safety Production University Key Laboratory of Sichuan Province, Sichuan Agricultural University, Chengdu, 611130 Sichuan China; 3 Key Laboratory of Animal Disease-Resistance Nutrition, Ministry of Education, Ministry of Agriculture and Rural Affairs, Key Laboratory of Sichuan Province, Chengdu, 611130 China

**Keywords:** *Ctenopharyngodon idella*, Digestive and absorptive capacity, FXR, Growth performance, Gut–liver axis, Hepatic lipid metabolism, Rosemary-derived triterpene acids

## Abstract

**Background:**

Rosemary-derived triterpene acids (TAs), primarily composed of ursolic acid, oleanolic acid, and betulinic acid, exhibit multiple bioactive properties. However, their effects on lipid metabolism and the underlying regulatory pathways remain unclear. This study investigated the effects of dietary supplementation with TAs on the growth performance, digestive and absorptive function, and hepatic lipid metabolism in juvenile grass carp (*Ctenopharyngodon idella*).

**Methods:**

In this trial, 2,160 juvenile grass carp (average weight 13.04 ± 0.02 g) were randomly allocated to six dietary treatments, each comprising six replicates with 60 fish per replicate. Fish were fed diets supplemented with increasing concentrations of TAs (0, 58.80, 179.30, 261.90, 312.00, and 390.00 mg/kg) for 70 d. At the end of the trial, relevant samples were collected for subsequent analyses.

**Results:**

The results demonstrated that dietary supplementation with TAs significantly increased specific growth rate (SGR), whole-body crude protein (CP) levels, and protein retention value (PRV) in juvenile grass carp, while reducing whole-body ether extract (EE) levels. Moreover, dietary supplementation with TAs significantly enhanced the activities of intestinal digestive enzymes and brush-border enzymes, thereby improving the digestive and absorptive capacity of juvenile grass carp. In the liver, dietary supplementation with TAs markedly inhibited lipid synthesis while promoting lipid utilization. The effects of TAs on lipid metabolism were associated with activation of the hepatic farnesoid X receptor (FXR) pathway, involving peroxisome proliferator-activated receptor alpha (PPARα) and sterol regulatory element-binding protein 1 (SREBP-1). Furthermore, TAs modulated the gut–liver axis by inhibiting the intestinal FXR–sphingomyelin phosphodiesterase 3 (SMPD3)–ceramide pathway, which may contribute to reduced hepatic lipid deposition. Quadratic regression analysis showed that the optimal dietary TAs supplementation levels were 245.00 mg/kg (SGR), 218.33 mg/kg (intestinal lipase activity), and 267.64 mg/kg (hepatic hormone-sensitive lipase activity).

**Conclusions:**

The addition of TAs to the diet improved growth performance, digestive and absorptive capacity, and liver lipid utilization in juvenile grass carp. This work reveals the potential application of TAs in aquaculture and provides a theoretical basis for the development of functional feed additives.

**Supplementary Information:**

The online version contains supplementary material available at 10.1186/s40104-025-01351-1.

## Introduction

With the continuous growth of the global population and the rising demand for high-quality animal protein, aquaculture has become one of the fastest-growing sectors in agriculture [1]. To meet increasing production demands, aquaculture is progressively shifting toward high-density, intensive farming systems [2]. However, intensive farming often involves overfeeding, which can disrupt the metabolic balance of fish, particularly lipid metabolism [3, 4]. For example, overfeeding impairs energy metabolism, leading to liver hypertrophy and damage in rainbow trout (*Oncorhynchus mykiss*) [5]. Plant extracts have attracted widespread attention as a potential strategy for improving aquaculture performance because of their natural safety and multiple bioactivities [6, 7]. Rosemary (*Rosmarinus officinalis *L.), a member of the Lamiaceae family, is widely used as a culinary herb and in traditional medicine [8]. Studies have shown that rosemary extract exerts hepatoprotective effects, which are mainly attributed to its bioactive compounds, such as triterpene acids, polyphenols, and diterpenes [9]. Among these, triterpene acids such as ursolic acid, oleanolic acid, and betulinic acid stand out for their structural similarity to steroid hormones and cholesterol, which makes them particularly relevant in modulating lipid metabolism [10]. Previous studies have reported that ursolic acid can improve hepatic lipid metabolism by regulating the intestinal microbiota in mice [11], and oleanolic acid can reduce lipid accumulation in the rat liver [12]. However, in practical production, the high extraction cost of single active compounds from plants limits their application in animal nutrition [13]. Extracts obtained from rosemary, which are rich in ursolic acid, oleanolic acid, and betulinic acid, may serve as a cost-effective alternative with synergistic advantages over individual triterpene acids. Nevertheless, the effects and mechanisms by which rosemary-derived triterpene acids (TAs) regulate lipid metabolism in animals remain unclear and warrant further investigation.

The liver, as the main site of lipid turnover, integrates the production, utilization, transport and deposition of lipids [14]. Farnesoid X receptor (FXR), a bile acid-activated nuclear receptor, is crucial for maintaining hepatic lipid homeostasis [15]. In the liver, FXR activation induces small heterodimer partner (SHP) expression, which inhibits sterol regulatory element-binding protein 1 (SREBP-1)-mediated lipogenesis and alleviates lipid accumulation [16]. FXR also promotes fatty acid β-oxidation by upregulating peroxisome proliferator-activated receptor alpha (PPARα) and its targets such as carnitine palmitoyltransferase 1A (CPT1A) [17]. Studies in fish have also demonstrated that activation of hepatic FXR promotes lipid metabolism [18, 19]. Triterpene acids are composed of six isoprene units and share structural features with bile acids and cholesterol, suggesting they may have similar biological functions [10]. It has been reported that betulinic acid can activate peroxisome proliferator-activated receptor γ coactivator 1 alpha (PGC-1α), a key coactivator of FXR, in brown adipocytes [20, 21]. Therefore, TAs may influence lipid metabolism by activating the hepatic FXR pathway.

In addition to the liver, the intestine can also regulate lipid metabolism via the gut–liver axis [22]. Through this axis, small molecules and bile acids are delivered to the liver via the bloodstream, affecting hepatic metabolic functions [23]. Interestingly, FXR in the intestine can affect lipid metabolism via the gut–liver axis in a manner opposite to that in the liver [24]. FXR activation in the liver promotes lipid utilization, whereas activation in the intestine leads to lipid accumulation in the liver by promoting ceramide secretion [25]. The accumulation of ceramide in the intestine disrupts hepatic lipid regulation via the gut–liver axis, resulting in lipid deposition and metabolic disorders [26]. A previous study found that F6, a derivative of betulinic acid, can alleviate hepatic lipid deposition by inhibiting intestinal FXR in mice [27]. The above studies suggest that TAs might regulate FXR in a tissue-specific manner, although further investigation is required to confirm this potential mechanism.

Grass carp (*Ctenopharyngodon idella*) is known for its rapid growth and desirable flavor, and currently ranks first in global freshwater aquaculture production [28]. This research investigated the impacts of dietary TAs on growth performance, hepatic FXR signaling pathway, and intestinal FXR–sphingomyelin phosphodiesterase 3 (SMPD3)–ceramide axis in grass carp, with the aim of elucidating the possible mechanism by which TAs regulate lipid metabolism. Therefore, this study is the first to evaluate the optimal addition level of TAs in grass carp, providing a scientific foundation for the advancement of novel functional feed additives.

## Materials and methods

### Animal ethics statement

All animal husbandry procedures in this experiment were approved by the Animal Care Advisory Committee of Sichuan Agricultural University (No. WZX-2023214080).

### Diets, animals, and experimental design

Table 1 presents the detailed formulation of the experimental diets. Protein sources primarily included fishmeal, dehulled soybean meal, rapeseed meal, and degossypolized cottonseed protein, while fats were mainly supplied by fish oil and soybean oil. The nutritional requirements for juvenile grass carp were based on those adopted in our previous laboratory study [30]. The nutritional composition of whole fish and experimental diets was analyzed according to AOAC methods [31]. The whole fish and experimental diets were dried at 105 °C and then analyzed as follows: protein by the Kjeldahl method (No. 990.03), crude fat by the Soxhlet extraction method (No. 2003.05), and ash by muffle furnace method (No. 923.03). The TAs used were provided as a crude extract of rosemary and were obtained from Geneham Pharmaceutical Co., Ltd. (Hunan, China). The crude rosemary extract contained 41.15% TAs (with ursolic acid, oleanolic acid, and betulinic acid present at 25.71%, 8.75%, and 6.69% of the crude rosemary extract, respectively). This crude rosemary extract was blended with microcrystalline cellulose to prepare premixes in which each kilogram contained 0.00, 6.48, 12.96, 19.44, 25.92, 32.40 g rosemary extract for treatments 1–6, respectively. These premixes were then incorporated into the experimental diets to achieve dietary TAs concentrations of 0.00 (basal diet), 80, 160, 240, 320, and 400 mg/kg, respectively. The concentrations of TAs in the experimental diets were determined by high-performance liquid chromatography (HPLC) using purified ursolic acid, oleanolic acid, and betulinic acid as standards for quantification. The TAs content was calculated as the sum of the measured values of these three components. HPLC analysis showed that the actual dietary concentrations of TAs were 0 (Below the detection limit), 58.80, 179.30, 261.90, 312.00, and 390.00 mg/kg. The diets were processed into pellets with a diameter of 2 mm according to the method of Shi et al. [32] and stored at −20 °C.

**Table 1:** Ingredients and nutrient content of the basal diet (air-dried basis)

Ingredients	g/kg	Nutrient content	g/kg
Fish meal (CP 67.95%)^1^	50.00	Moisture^6^	102.22
Dehulled soybean meal (CP 46.54%)^1^	210.00	Dry matter^6^	897.78
Degossypolized cottonseed protein (CP 65.73%)^1^	200.00	Crude protein^6^	318.42
Rapeseed meal (CP 38.48%)^1^	130.50	Crude lipid^6^	49.03
Fish oil (EE 99.90%)^1^	25.70	Ash^6^	71.71
Soybean oil (EE 99.00%)^1^	12.60	Crude fiber^6^	68.53
α-Starch	259.55	Nitrogen free extract^6^	390.09
Ca(H_2_PO_4_)_2_	34.40	Gross energy^6^, MJ/kg	16.92
Vitamin premix^2^	20.00	Available phosphorus^7^	8.40
Mineral premix^3^	10.00	n-3 PUFA^7^	10.40
Choline chloride premix^4^	10.00	n-6 PUFA^7^	9.60
Butylated hydroxyanisole	0.15		
L-Lysine hydrochloride (78.8%)	4.00		
L-Threonin (98.5%)	3.10		
TAs premix ^5^	30.00		
Total	1,000.00		

^1^Peruvian fishmeal (Pesquera Exalmar S.A.A., Lima, Peru), dehulled soybean meal (COFCO Oils & Grains Industries (Chongqing) Co., Ltd., Chongqing, China), rapeseed meal (Ausca OILS and Grains Industries Co., Ltd., Fangchenggang, China), degossypolized cottonseed protein (Chenguang Biotech Group Co., Ltd., Bazhou, China), fish oil (Damao Grain and Oil Co., Ltd., Foshan, China), and soybean oil (Litai Grain and Oil Co., Ltd., Chengdu, China). The crude protein and crude lipid contents in feed ingredients are the actual measured values

^2^Per kilogram of vitamin premix (g/kg): retinyl acetate (500,000 IU/g), 0.386 g; cholecalciferol (500,000 IU/g), 0.277 g; DL-α-tocopherol acetate (50%), 43.903 g; menadione (50%), 0.380 g; thiamin nitrate (98%), 0.164 g; riboflavin (80%), 0.775 g; pyridoxine hydrochloride (98%), 0.753 g; D-calcium pantothenate (98%), 4.203 g; niacinamide (99%), 2.576 g; inositol (97%), 22.062 g; VB_12_ (1%), 0.94 g; D-biotin (2%), 1.550 g; folate acid (95%), 0.379 g; VC (95%), 16.316 g. All ingredients were filled with corn starch to 1 kg

^3^Per kilogram of mineral premix (g/kg): Ferrous fumarate (30.6% Fe), 12.361 g; CuSO_4_·5H_2_O (25.1% Cu), 0.952 g; ZnSO_4_·H_2_O (34.5% Zn), 7.681 g; MnSO_4_·H_2_O (31.8% Mn), 3.066 g; Ca(IO_3_)_2_ (3.2% I), 1.563 g; Yeast selenium (0.2%), 13.650 g; MgSO_4_·H_2_O (15.0% Mg), 256.793 g. All ingredients were filled with corn starch to 1 kg

^4^Per kilogram of choline chloride premix (g/kg): Choline chloride (50%), 306.71 g; corn starch, 693.29 g

^5^Per kilogram of TAs premix (g/kg): from treatment 1 to 6, the supplementation of crude rosemary extract (411.50 g TAs/kg) was 0.00, 6.48, 12.96, 19.44, 25.92, 32.40 g, respectively, and the rest was filled with microcrystalline cellulose

^6^Moisture, dry matter, crude protein, crude lipid, ash, crude fiber, and gross energy contents were measured values, while nitrogen free extract content was calculated based on the above results

^7^Available phosphorus, n-3 PUFA, and n-6 PUFA contents were calculated according to NRC [29]

### Experimental conditions and feeding management

The juvenile grass carp used in the experiment were obtained from a farm in Deyang (Sichuan Province, China). This experiment was conducted at the Aquatic Animal Nutrition Experimental Base, Animal Nutrition Institute, Sichuan Agricultural University. Prior to the feeding trial, juvenile grass carp were placed in experimental net cages (1.4 m × 1.4 m × 1.4 m) installed in the experimental ponds for a temporary rearing period of 4 weeks [30]. During the temporary rearing period, juvenile grass carp were fed a commercial diet (Sichuan Kefei Feed Technology Co., Ltd., Deyang, China) four times daily at 07:00, 11:00, 15:00, and 19:00 to acclimate them to the experimental conditions. Then, 2,160 juvenile grass carp (13.04 ± 0.02 g) with normal body coloration, vigorous activity, and uniform body size were randomly allocated to six groups, each comprising six replicates with 60 fish per replicate. Water was supplied from surrounding rivers and was first settled and disinfected in a sedimentation pond before being delivered to the experimental ponds. Throughout the trial period, microporous aeration was used and the water was changed daily, thereby ensuring that water quality remained within appropriate parameters. Water temperature, pH, and dissolved oxygen (DO) were monitored daily and averaged 30.8 ± 1.5 °C, 7.6 ± 0.2, and 6.5 ± 0.5 mg/L, respectively. The growth trial lasted 70 d, during which juvenile grass carp were fed to satiation four times daily (07:00, 11:00, 15:00, and 19:00).

### Sample collection

Initial and final weights of fish in each net were recorded to calculate growth performance. Before sampling, fish were anesthetized with 50 mg/kg MS-222. At the start of the trial, 6 fish were sampled to determine the initial whole-body nutrient composition, and 6 fish per treatment were sampled at the end of the trial to assess the final whole-body nutrient composition. Moreover, at the end of the trial, 60 fish per treatment were sampled for serum and tissue (liver and intestine) collection. Tissue samples from 48 fish per group were immediately stored at −80 °C for biochemical analyses. Tissue samples from the remaining 12 fish per group were divided into two portions, with one portion fixed in 4% paraformaldehyde and the other stored at −80 °C [33]. Paraformaldehyde-fixed liver samples were used for histological and immunofluorescence analyses, whereas the corresponding intestinal samples were used for immunofluorescence analyses. Tail vein blood was collected using non-heparinized syringes and centrifuged at 1,000 × *g* for 15 min at 4 °C to obtain serum, which was then stored at −20 °C for later analysis. The subsequent analyses were performed using six biological replicates per treatment [34].

### Growth performance, somatic index and nutrient retention rate index analysis

The specific growth rate (SGR), feed intake (FI), feed efficiency (FE), feed conversion ratio (FCR), intestinosomatic index (ISI), hepatosomatic index (HSI), protein efficiency ratio (PER), protein retention value (PRV), lipid retention value (LRV), and ash retention value (ARV) of fish were calculated as follows:

$$\mathrm{SGR}\;\left(\%/\mathrm d\right)\;=\;\left[\ln\left(\mathrm{FBW}\left(\mathrm g/\mathrm{fish}\right)\right)\;-\;\ln\left(\mathrm{IBW}\;\left(\mathrm g/\mathrm{fish}\right)\right)\right]/\mathrm{days}\;\times\;100$$

$$\mathrm{FI}\left(\mathrm g/\mathrm{fish}\right)=\mathrm{total}\;\mathrm{feed}\;\mathrm{consumption}\;\left(\mathrm g/\mathrm{fish}\right)-\mathrm{total}\;\mathrm{uneaten}\;\mathrm{feed}\left(\mathrm g/\mathrm{fish}\right)$$

$$\mathrm{FE}=\left[\mathrm{FBW}\left(\mathrm g/\mathrm{fish}\right)-\mathrm{IBW}\left(\mathrm g/\mathrm{fish}\right)\right]/\mathrm{FI}\left(\mathrm g/\mathrm{fish}\right)$$

$$\mathrm{FCR}=\mathrm{FI\left(g/fish\right)/\left[FBW\left(g/fish\right)-IBW\left(g/fish\right)\right]}$$

$$\mathrm{ISI}\left(\%\right)=\left[\text{intestinal weight}\left(\mathrm{g}\right)/\text{body weight}\left(\mathrm{g}\right)\right]\times100$$

$$\mathrm{HSI}\left(\mathrm{\%}\right)=\left[\mathrm{liver}\;\mathrm{weight}\left(\mathrm g\right)/\mathrm{body}\;\mathrm{weight}\left(\mathrm g\right)\right]\times100$$

$$\mathrm{PER}=\left[\mathrm{FBW}\left(\mathrm g/\mathrm{fish}\right)-\mathrm{IBW}\left(\mathrm g/\mathrm{fish}\right)\right]/\mathrm{protein}\;\mathrm{intake}\left(\mathrm g/\mathrm{fish}\right)$$

$$\mathrm{PRV}\left(\mathrm{\%}\right)=\left[\mathrm{fish}\;\mathrm{protein}\;\mathrm{gain}\left(\mathrm g\right)/\mathrm{protein}\;\mathrm{intake}\left(\mathrm g\right)\right]\times100$$

$$\mathrm{LRV}\left(\%\right)=\left[\mathrm{fish}\;\mathrm{lipid}\;\mathrm{gain}\;\left(\mathrm g\right)/\mathrm{lipid}\;\mathrm{intake}\left(\mathrm g\right)\right]\times100$$

$$\mathrm{ARV}\left(\mathrm{\%}\right)=\left[\mathrm{fish}\;\mathrm{ash}\;\mathrm{gain}\left(\mathrm g\right)/\mathrm{ash}\;\mathrm{intake}\left(\mathrm g\right)\right]\times100$$

In the above formulas, IBW and FBW represent the initial and final body weights, respectively.

### Histological observations

Histological observation procedures followed the method described by He et al. [35]. A portion of the liver was collected from fish and immediately fixed in 4% paraformaldehyde. The tissues were then gradually dehydrated in graded concentrations of ethanol (75%, 85%, 95%, and 100%) and embedded in paraffin. Paraffin-embedded tissues were sectioned using a Leica HistoCore MULTICUT microtome (Leica, Germany). Liver sections were stained with hematoxylin–eosin (H&E) and Oil Red O by Chengdu Lilai Biotechnology Co., Ltd. (Chengdu, China), and analyzed under an Olympus BX43 light microscope (Olympus Corporation, Tokyo, Japan) at 200 × or 400 × magnification.

### Biochemical parameters in serum, enzyme activities and ceramide contents in the intestine and liver

Intestinal and liver tissues were ground and centrifuged with saline at a ratio of 1:9 to prepare 10% tissue homogenates, which were used for subsequent assays. Serum biochemical parameters, including triglyceride (TG), total cholesterol (TC), low-density lipoprotein cholesterol (LDL-C), high-density lipoprotein cholesterol (HDL-C), glutamate pyruvate transaminase (GPT), and glutamate oxaloacetate transaminase (GOT), were measured. Biochemical parameters related to intestinal digestion and absorption function included trypsin, chymotrypsin, lipase, amylase, alkaline phosphatase (AKP), sodium–potassium adenosine triphosphatase (Na⁺/K⁺-ATPase), gamma-glutamyl transferase (γ-GT), and creatine kinase (CK). Liver lipid metabolism-related parameters included fatty acid synthase (FAS), adipose triglyceride lipase (ATGL), and hormone-sensitive lipase (HSL). All of the above kits were obtained from Nanjing Jiancheng Bioengineering Institute (Nanjing, China), and the specific methods were described in the instruction manual. In addition, liver and intestinal ceramide contents were analyzed with kits supplied by Shanghai Enzyme-linked Biotechnology Co., Ltd. (Shanghai, China) according to the manufacturer’s protocols.

### Molecular docking

Molecular docking of ursolic acid, oleanolic acid and betulinic acid with FXR was carried out using AutoDock QVina-W. Docking results were analyzed using AutoDock and PyMOL Open Source Edition software and images were drawn using PyMol Open Source Edition. The 3D structure SDF files of ursolic acid, oleanolic acid, and betulinic acid were obtained from the PubChem database, converted to PDB format, energy minimized on the Yinfu Cloud Computing Platform (https://cloud.yinfotek.com/), and exported as ligand PDBQT files using AutoDock software. The FXR protein structure (ID = 6HL0) was downloaded from the RCSB Protein Data Bank, imported into spdbv software to repair incomplete regions, and further processed in PyMOL Open Source Edition to remove non-essential molecules and prepare the complete protein structure. The equilibrated protein was then prepared in AutoDock software by adjusting charges and adding hydrogen atoms and exported as a receptor PDBQT file. First, QVina-W was utilized to perform blind docking of individual ligands with FXR to identify potential binding sites. The blind docking results were used to configure the protein docking box, generate a Vina configuration file, and perform further docking using QVina-W to select the best binding poses for analysis.

### Real-time quantitative PCR analysis

Liver and intestinal tissues were ground on ice and total RNA was extracted from the tissue homogenate using the TRIzol method [36]. RNA purity and concentration were respectively assessed via agarose gel electrophoresis and NanoDrop 2000 spectrophotometer (Thermo Scientific, USA). Reverse transcription was conducted with the TaKaRa PrimeScript^®^ RT kit. The primers used in this experiment were partly adopted from previous publications and partly designed in our laboratory. The reaction system for qPCR contained 1 µL complementary DNA, 0.2 µL ROX, 5 µL SYBR Premix Ex Taq, 0.4 µL primers, and 3.4 µL nuclease-free water (Vazyme, Nanjing, China). The reaction was run in a QuantStudio™ 5 Real-Time PCR System (Life Technologies, Carlsbad, CA, USA) according to the manufacturer’s protocol. The cycling conditions were as follows: 95 °C for 2 min, followed by 40 cycles of 95 °C for 15 s and 60 °C for 30 s. β-Actin and *GAPDH* were selected as the reference genes, and relative expression levels of target genes were calculated using the 2^−ΔΔCt^ method. Primer sequences are provided in Table S1.

### Western blot analysis

PMSF was prepared with RIPA (Beyotime, China) at a ratio of 10 μL:800 μL for extraction of liver and intestinal tissue proteins. Following Chen et al. [37], target proteins were separated by 8% SDS-PAGE and transferred onto PVDF membranes. After blocking, the PVDF membranes were incubated with primary antibodies at 4 °C for 16 h, followed by incubation with secondary antibodies at room temperature for 1 h. Protein bands were detected using the ECL kit (Beyotime, China) and visualized with the Touch Imager Pro (eBlot, China). Band intensities were quantified using ImageJ 1.53 K, with GAPDH as the internal reference for normalization. Details of the primary antibodies used are provided in Table S2.

### Immunofluorescence analysis

The paraffin embedding and sectioning methods for intestinal tissue were consistent with those described for liver tissue in Section "Histological observations". The sections of liver and intestinal tissue were deparaffinized, blocked for endogenous peroxidase activity, and subjected to antigen retrieval [38]. The samples were first incubated with primary antibodies at 4 °C for 16 h, followed by a 1 h incubation at room temperature protected from light with anti-rabbit IgG (Servicebio, China). Nuclei were then stained using antifade mounting medium with DAPI (Servicebio, China). An inverted fluorescence microscope (Leica DMI8, Germany) was used to examine the samples, and fluorescence signals were quantified using ImageJ. The information about the primary antibodies used for immunofluorescence is detailed in Table S3.

### Statistical analysis

Statistical analyses were performed with SPSS 27.0. After testing for normality, the data were analyzed by one-way ANOVA, and Duncan’s multiple range test was used to compare differences among the TAs treatment groups. Data are expressed as mean ± standard deviation (SD), and differences were considered significant at *P* < 0.05. Orthogonal polynomial contrasts were used to evaluate linear and quadratic dose–response relationships. Comparisons between linear and quadratic regression models were based on the coefficient of determination (*R*^2^) to determine the appropriate dietary supplementation levels of TAs.

## Results

### Effects of TAs on growth performance and whole-body proximate compositions

As shown in Table 2, compared with the 0.00 mg/kg TAs group, dietary supplementation with 58.80–390.00 mg/kg TAs significantly increased the FBW, SGR, FI, and HSI of juvenile grass carp, all of which peaked in the 261.90 mg/kg TAs group (*P* < 0.05). Furthermore, compared with the 0.00 mg/kg TAs group, dietary supplementation with 261.90 mg/kg TAs significantly increased PRV and whole-body crude protein (CP) content, while decreasing whole-body moisture and ether extract (EE) contents of juvenile grass carp (*P* < 0.05). Although no significant differences were observed among treatments (*P* > 0.05), quadratic regression analysis revealed a significant quadratic relationship between LRV and dietary TA levels (*P* < 0.05). Dietary TAs had no significant effects on the FE, FCR, ISI, PER, ARV, or whole-body ash content (*P* > 0.05).

**Table 2:** Effects of TAs on the growth performance, whole-body nutritional components, and nutrient conversion rates of juvenile grass carp

Item	**Dietary TA levels, mg/kg**						***P*-value**		
	**0.00**	**58.80**	**179.30**	**261.90**	**312.00**	**390.00**	**ANOVA**	**Linear**	**Quadratic**
Growth performance^1^									
IBW, g/fish	13.05 ± 0.02	13.04 ± 0.01	13.04 ± 0.02	13.04 ± 0.02	13.03 ± 0.02	13.04 ± 0.02	0.738	0.264	0.965
FBW, g/fish	200.56 ± 5.13^a^	250.70 ± 5.89^b^	284.80 ± 5.20^c^	306.88 ± 15.17^d^	283.07 ± 8.08^c^	249.95 ± 5.22^b^	< 0.001	< 0.001	< 0.001
SGR, %/d	3.90 ± 0.04^a^	4.22 ± 0.03^b^	4.41 ± 0.03^c^	4.51 ± 0.07^d^	4.40 ± 0.04^c^	4.22 ± 0.03^b^	< 0.001	< 0.001	< 0.001
FI, g/fish	169.80 ± 0.00^a^	215.95 ± 0.00^c^	247.06 ± 0.01^e^	267.83 ± 0.44^f^	245.25 ± 0.15^d^	214.82 ± 0.01^b^	< 0.001	< 0.001	< 0.001
FE	1.10 ± 0.03	1.10 ± 0.03	1.10 ± 0.02	1.10 ± 0.05	1.10 ± 0.03	1.10 ± 0.02	0.999	1.000	0.697
FCR	0.91 ± 0.02	0.91 ± 0.02	0.91 ± 0.02	0.91 ± 0.05	0.91 ± 0.03	0.91 ± 0.02	0.999	0.962	0.740
ISI, %	2.58 ± 0.28	2.65 ± 0.10	2.67 ± 0.12	2.78 ± 0.18	2.70 ± 0.18	2.67 ± 0.05	0.441	0.207	0.146
HSI, %	1.44 ± 0.14^a^	1.65 ± 0.16^b^	1.85 ± 0.13^b^	2.06 ± 0.15^c^	1.76 ± 0.24^b^	1.76 ± 0.15^b^	< 0.001	< 0.001	< 0.001
Whole-body nutritional components^2^									
Moisture, %	77.53 ± 0.97^b^	76.12 ± 1.92^ab^	77.25 ± 0.81^b^	75.40 ± 0.70^a^	76.45 ± 0.64^ab^	77.03 ± 1.13^b^	0.024	0.388	0.027
CP, %	11.75 ± 0.99^a^	12.85 ± 0.79^b^	12.75 ± 0.80^b^	13.42 ± 0.50^b^	12.92 ± 0.25^b^	12.58 ± 0.63^ab^	0.011	0.045	0.002
EE, %	10.46 ± 0.62^b^	10.13 ± 0.78^b^	9.12 ± 0.27^a^	8.79 ± 0.55^a^	9.37 ± 0.38^a^	9.22 ± 0.51^a^	< 0.001	< 0.001	0.001
Ash, %	2.90 ± 0.16	2.92 ± 0.21	2.82 ± 0.16	2.93 ± 0.18	2.89 ± 0.12	2.84 ± 0.27	0.905	0.667	0.886
Nutrient conversion rates^2^									
PER	3.47 ± 0.09	3.49 ± 0.09	3.52 ± 0.07	3.48 ± 0.17	3.47 ± 0.10	3.51 ± 0.08	0.969	0.715	0.962
PRV, %	40.82 ± 5.82^a^	39.60 ± 5.60^a^	40.35 ± 5.30^a^	49.44 ± 2.60^b^	42.42 ± 5.79^a^	43.87 ± 4.39^ab^	0.023	0.066	0.356
LRV, %	240.85 ± 45.00	204.68 ± 34.42	187.82 ± 26.18	204.70 ± 12.32	199.00 ± 25.19	207.30 ± 25.63	0.088	0.110	0.023
ARV, %	45.01 ± 5.31	40.11 ± 5.46	39.65 ± 7.35	46.82 ± 3.02	40.89 ± 5.56	42.84 ± 6.77	0.216	0.948	0.570

*IBW* Initial body weight, *FBW* Final body weight, *SGR* Specific growth rate, *FI* Feed intake, *FE* Feed efficiency, *FCR* Feed conversion ratio, *ISI* Intestinosomatic index, *HSI* Hepatosomatic index, *CP* Crude protein, *EE* Ether extract, *PER* Protein efficiency, *PRV* Protein retention value, *LRV* Lipid retention value, *ARV* Ash retention value

Results were expressed as mean ± SD; ^a^^–^^f^ Mean values within a row with different superscript letters indicate significant different (*P* < 0.05)

^1^*n* = 6 replicates per treatment, with 60 fish in each replicate

^2^*n* = 6 fish per treatment

### Absorption and metabolism of TAs

**Fig. 1:**
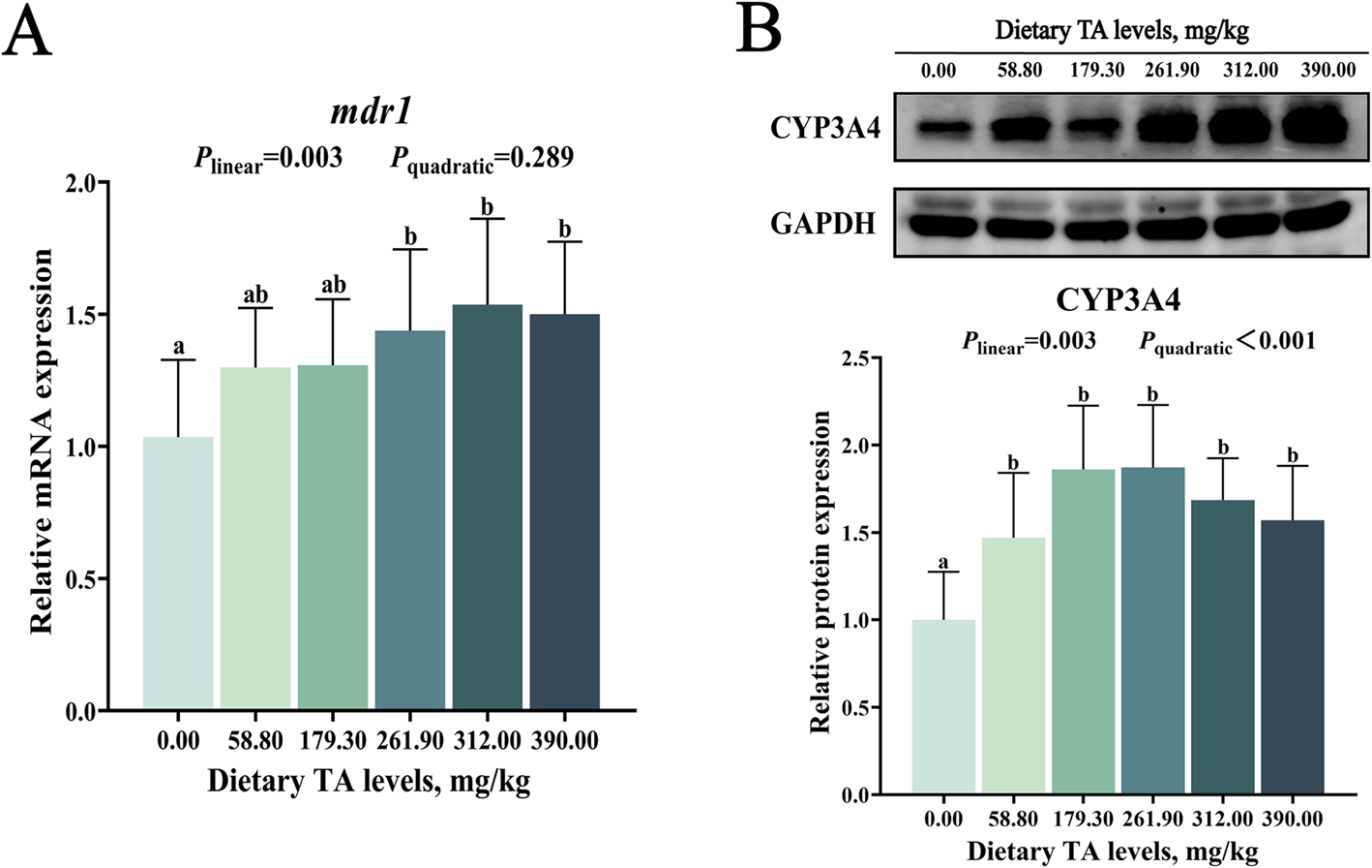
Effects of TAs on intestinal absorption and hepatic metabolism of TAs. **A** The mRNA expression of *mdr1* in grass carp intestine. **B** The protein levels of CYP3A4 in grass carp liver. Results were expressed as mean with SD (*n* = 6). The columns are marked with different letters to indicate significant differences (*P* < 0.05)

As illustrated in Fig. 1, compared with the 0.00 mg/kg TAs group, dietary supplementation with 261.90–390.00 mg/kg TAs significantly increased the relative intestinal mRNA expression levels of multidrug resistance 1 (*mdr1*), peaking at 312.00 mg/kg TAs (*P* < 0.05). Furthermore, compared with the 0.00 mg/kg TAs group, dietary supplementation with 58.80–390.00 mg/kg TAs significantly increased the relative hepatic protein levels of cytochrome P450 3A4 (CYP3A4), with the highest level observed at 261.90 mg/kg TAs (*P* < 0.05).

### Effects of TAs on intestinal digestive and absorptive enzymes

The influence of dietary TAs on intestinal digestive and brush border enzymes is presented in Table 3. Compared with the 0.00 mg/kg TAs group, supplementation with 179.30–312.00 mg/kg TAs in the diet significantly increased intestinal activities of trypsin, chymotrypsin, lipase, amylase, AKP, Na⁺/K⁺-ATPase, γ-GT, and CK (*P* < 0.05), with peak activities observed in different TAs groups: trypsin and AKP at 179.30 mg/kg; lipase, γ-GT, and CK at 261.90 mg/kg; and chymotrypsin, amylase, and Na⁺/K⁺-ATPase at 312.00 mg/kg.

**Table 3:** Effects of TAs on the digestive enzymes and brush border enzymes of juvenile grass carp

Item	**Dietary TA levels,** **mg/kg**						***P*-value**		
	**0.00**	**58.80**	**179.30**	**261.90**	**312.00**	**390.00**	**ANOVA**	**Linear**	**Quadratic**
Digestive enzymes									
Trypsin, U/mg prot	2,184.90 ± 181.01^a^	2,679.97 ± 245.98^b^	4,701.67 ± 454.56^d^	4,666.20 ± 444.79^d^	3,834.55 ± 338.12^c^	2,337.91 ± 186.76^ab^	< 0.001	< 0.001	< 0.001
Chymotrypsin, U/mg prot	1.17 ± 0.10^a^	1.20 ± 0.10^a^	2.09 ± 0.18^b^	2.37 ± 0.25^c^	2.65 ± 0.18^d^	2.25 ± 0.22^bc^	< 0.001	< 0.001	< 0.001
Lipase, U/g prot	4.32 ± 0.40^a^	6.44 ± 0.35^b^	7.87 ± 0.63^c^	9.46 ± 0.50^d^	8.04 ± 0.51^c^	6.35 ± 0.54^b^	< 0.001	< 0.001	< 0.001
Amylase, U/mg prot	2.47 ± 0.13^a^	2.72 ± 0.08^b^	2.99 ± 0.09^d^	3.34 ± 0.12^e^	3.36 ± 0.14^e^	2.86 ± 0.09^c^	< 0.001	< 0.001	< 0.001
Brush border enzymes									
AKP, King unit/g prot	39.13 ± 2.77^a^	42.61 ± 3.22^a^	67.16 ± 7.41^d^	51.20 ± 4.14^c^	49.01 ± 5.30^bc^	44.80 ± 3.91^ab^	< 0.001	0.059	< 0.001
Na^+^/K^+^-ATPase, U/mg prot	0.31 ± 0.02^a^	0.42 ± 0.03^b^	0.47 ± 0.03^c^	0.50 ± 0.05^ cd^	0.52 ± 0.05^d^	0.40 ± 0.03^b^	< 0.001	< 0.001	< 0.001
γ-GT, U/g prot	18.40 ± 1.14^a^	19.20 ± 1.43^a^	26.12 ± 2.39^c^	34.21 ± 2.17^d^	26.77 ± 0.91^c^	22.10 ± 1.70^b^	< 0.001	< 0.001	< 0.001
CK, U/mg prot	0.57 ± 0.01^a^	0.66 ± 0.02^b^	0.83 ± 0.04^d^	0.85 ± 0.05^d^	0.74 ± 0.03^c^	0.63 ± 0.05^b^	< 0.001	< 0.001	< 0.001

*AKP* Alkaline phosphatase, *γ-GT *γ-Glutamyl transpeptidase, *CK* Creatine kinase

Results were expressed as mean ± SD (*n* = 6); ^a^^–^^e^ Mean values within a row with different superscript letters indicate significant different (*P* < 0.05 )

### Effects of TAs on liver health and body lipid metabolism

#### Histological observations of the liver

**Fig. 2:**
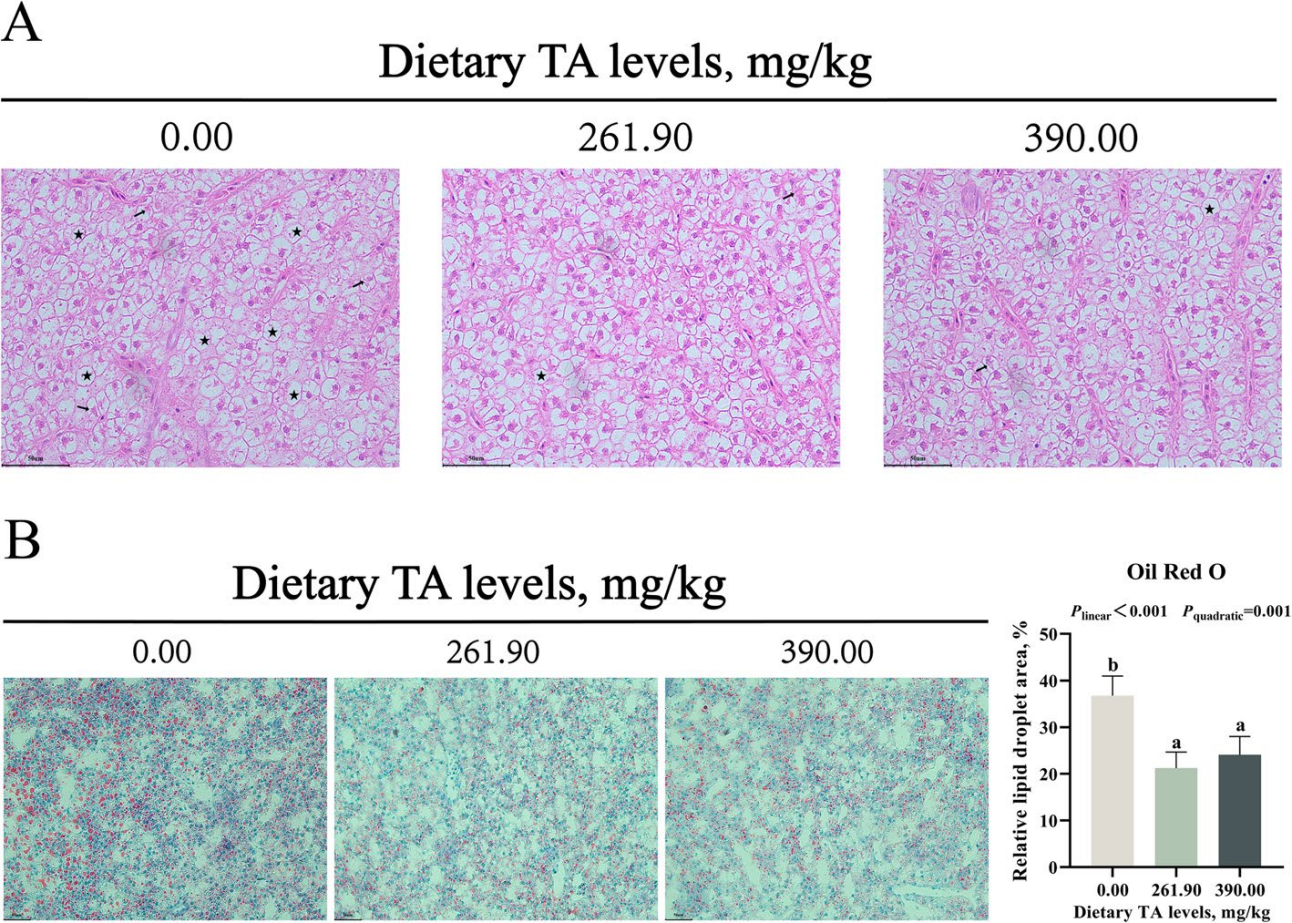
Effects of TAs on liver health and lipid metabolism. **A** Hematoxylin–eosin (H&E) staining of liver (scale bar = 50 μm, magnification 400 ×). Vacuolation (★). Karyorrhexis (black arrows). **B** and **C** Liver Oil Red O staining and its quantification (scale bar = 50 μm, magnification 200 ×). Results were expressed as mean with SD (*n* = 6). Different letters indicate significant difference (*P* < 0.05)

As shown in Fig. 2A, the results of H&E staining of liver tissue showed that hepatocytes in the 0.00 mg/kg TAs group exhibited a higher incidence of vacuolation and nuclear fragmentation, whereas liver cells in the 261.90 and 390.00 mg/kg TAs groups remained relatively intact. Moreover, as shown in Fig. 2B, the liver tissue of the 0.00 mg/kg TAs group contained numerous lipid droplets, while the lipid droplets in the liver of the 261.90 and 390.00 mg/kg TAs groups were significantly reduced (*P* < 0.05).

#### Effects of TAs on lipid metabolism–related serum biochemical parameters

As shown in Table 4, compared with the 0.00 mg/kg TAs group, dietary supplementation with 179.30–390.00 mg/kg TAs significantly decreased the activities of serum GPT and GOT, reaching the minimum in the 261.90 mg/kg TAs group (*P* < 0.05). Dietary supplementation with 261.90 mg/kg TAs also significantly reduced serum TG, TC, and LDL-C levels compared with the 0.00 mg/kg TAs group (*P* < 0.05). In addition, compared with the 0.00 mg/kg TAs group, dietary supplementation with 58.80–390.00 mg/kg TAs significantly increased serum HDL-C levels, with the maximum value observed in the 261.90 mg/kg TAs group (*P* < 0.05).

**Table 4:** Effects of TAs on serum and hepatic lipid metabolism and health biomarkers in juvenile grass carp

Item	**Dietary TA levels, mg/kg**						***P*-value**		
	**0.00**	**58.80**	**179.30**	**261.90**	**312.00**	**390.00**	**ANOVA**	**Linear**	**Quadratic**
Serum lipid metabolism and health									
GOT, U/L	12.96 ± 1.26^d^	10.43 ± 0.95^c^	10.06 ± 0.97^bc^	8.54 ± 0.78^a^	9.00 ± 1.03^ab^	9.56 ± 0.96^abc^	< 0.001	< 0.001	< 0.001
GPT, U/L	7.23 ± 0.50^c^	6.84 ± 0.74^bc^	6.28 ± 0.55^ab^	6.00 ± 0.57^a^	6.25 ± 0.36^ab^	6.26 ± 0.46^ab^	0.004	< 0.001	0.015
TG, mmol/L	1.78 ± 0.13^b^	1.71 ± 0.12^b^	1.63 ± 0.14^ab^	1.55 ± 0.12^a^	1.70 ± 0.13^ab^	1.72 ± 0.11^b^	0.048	0.351	0.006
TC, mmol/L	7.71 ± 0.57^d^	7.36 ± 0.60^ cd^	5.51 ± 0.32^b^	4.75 ± 0.35^a^	6.91 ± 0.55^c^	6.85 ± 0.52^c^	< 0.001	< 0.001	< 0.001
LDL-C, mmol/L	2.74 ± 0.27^b^	2.70 ± 0.23^b^	2.46 ± 0.24^a^	2.35 ± 0.17^a^	2.36 ± 0.12^a^	2.72 ± 0.16^b^	0.002	0.084	< 0.001
HDL-C, mmol/L	7.00 ± 0.60^a^	7.89 ± 0.43^b^	8.04 ± 0.68^b^	8.13 ± 0.61^b^	7.86 ± 0.72^b^	7.84 ± 0.53^b^	0.037	0.050	0.009
Liver lipid metabolism									
FAS, ng/mg prot	1.89 ± 0.23^e^	1.72 ± 0.04^d^	1.01 ± 0.09^c^	0.46 ± 0.05^b^	0.30 ± 0.02^a^	0.26 ± 0.02^a^	< 0.001	< 0.001	< 0.001
ATGL, U/g prot	431.68 ± 20.56^a^	476.43 ± 43.28^b^	586.10 ± 32.10^c^	597.96 ± 17.47^c^	680.02 ± 30.07^d^	437.26 ± 36.88^a^	< 0.001	< 0.001	< 0.001
HSL, ng/mg prot	1.41 ± 0.06^a^	1.52 ± 0.04^b^	1.58 ± 0.05^bc^	1.61 ± 0.07^c^	1.56 ± 0.05^bc^	1.58 ± 0.05^bc^	< 0.001	< 0.001	< 0.001

*GOT* Glutamate oxaloacetate transaminase, *GPT* Glutamate pyruvate transaminase, *TG* Triglycerides, *TC* Total cholesterol, *LDL-C* Low-density lipoprotein cholesterol, *HDL-C* High-density lipoprotein cholesterol, *FAS* Fatty acid synthase, *ATGL* Adipose Triglyceride Lipase, *HSL* Hormone-sensitive lipase

Results were expressed as mean ± SD (*n* = 6); ^a^^–^^e^ Mean values within a row with different superscript letters indicate significant different (*P* < 0.05)

### Effects of TAs on lipid metabolism and FXR signaling pathway in liver

The effects of TAs on hepatic lipid metabolism related enzymes are shown in Table 4. Compared with the 0.00 mg/kg TAs group, dietary supplementation with 58.80–390.00 mg/kg TAs significantly reduced hepatic FAS content, which dipped to its lowest level in the 390.00 mg/kg TAs group (*P* < 0.05). Moreover, compared with the 0.00 mg/kg TAs group, dietary supplementation with 58.80–312.00 mg/kg TAs increased hepatic ATGL activity and HSL content, peaking at 312.00 mg/kg and 261.90 mg/kg TAs, respectively (*P* < 0.05).

**Fig. 3:**
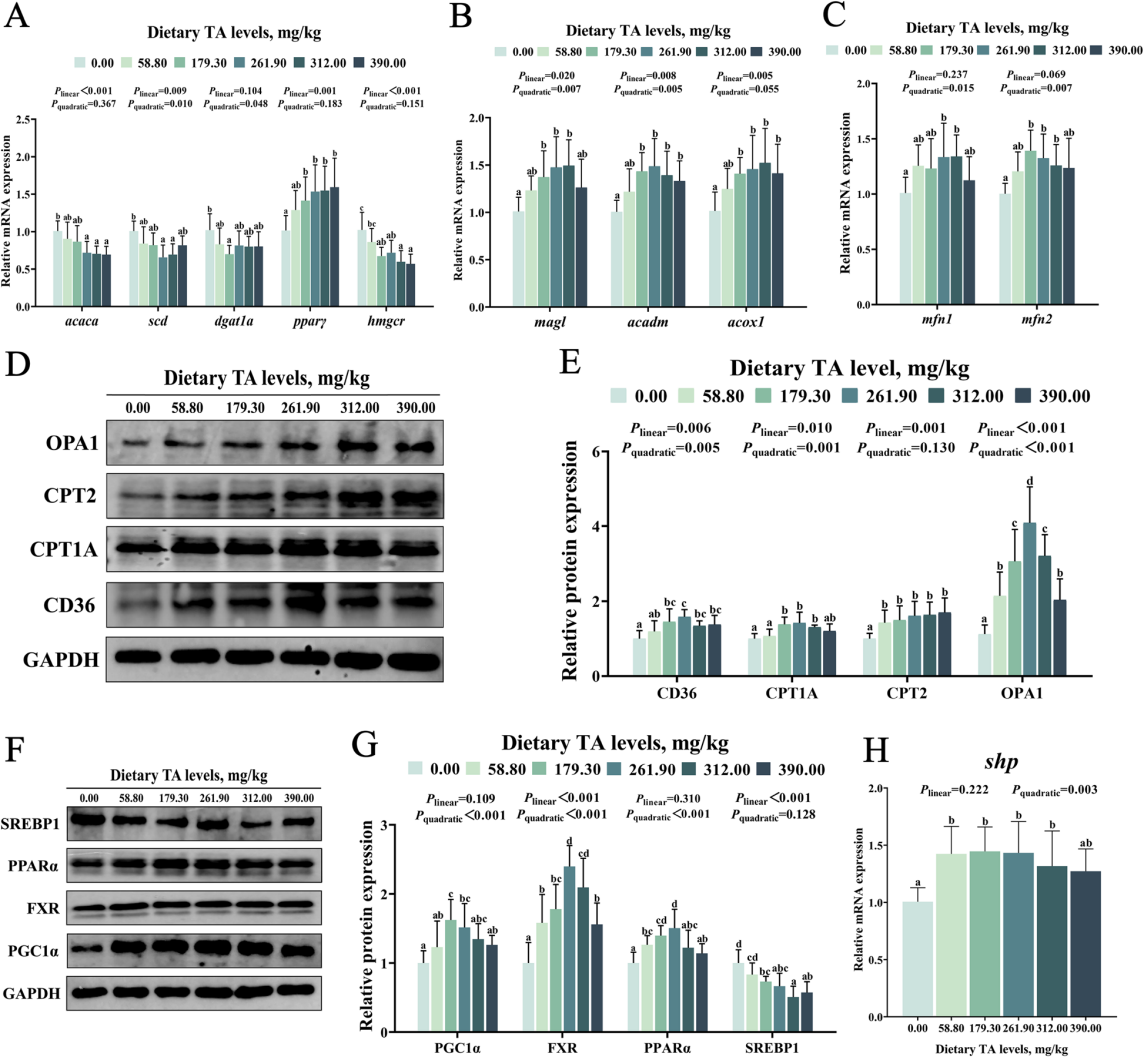
Effects of TAs on liver lipid metabolism and FXR pathway. **A **and **B** The mRNA expression of lipid synthesis and catabolism-related genes in grass carp liver. **C** The mRNA expression of *mfn1 *and *mfn2* in grass carp liver. **D **and **E** The protein levels of CD36, CPT1A, CPT2 and OPA1 in grass carp liver. **F **and **G** The protein levels of PGC-1α, FXR, PPARα and SREBP-1 in grass carp liver. **H** The mRNA expression of *shp* in grass carp liver. Results were expressed as mean with SD (*n* = 6). Different letters indicate significant difference (*P* < 0.05)

According to Fig. 3A, compared with the 0.00 mg/kg TAs group, dietary supplementation with 261.90–312.00 mg/kg TAs significantly suppressed the mRNA expression levels of hepatic acetyl-CoA carboxylase alpha (*acaca*), stearoyl-CoA desaturase (*scd*) and 3-hydroxy-3-methylglutaryl-CoA reductase (*hmgcr*), reaching their lowest levels at 390.00, 261.90 and 390.00 mg/kg TAs, respectively (*P* < 0.05). Similarly, compared with the 0.00 mg/kg TAs group, dietary supplementation with 179.30 mg/kg TAs significantly reduced the hepatic diacylglycerol O-acyltransferase 1a (*dgat1a*) mRNA expression level (*P* < 0.05, Fig. 3A). In contrast, compared with the 0.00 mg/kg TAs group, dietary supplementation with 179.30–312.00 mg/kg TAs significantly increased the mRNA expression levels of hepatic peroxisome proliferator-activated receptor gamma (*pparγ*), monoacylglycerol lipase (*magl*), medium-chain acyl-CoA dehydrogenase (*acadm*), and acyl-CoA oxidase 1 (*acox1*), reaching their peaks at 261.90–390.00 mg/kg TAs (*P* < 0.05, Fig. 3A and B). As illustrated in Fig. 3D and E, compared with the 0.00 mg/kg TAs group, dietary supplementation with 179.30–312.00 mg/kg TAs significantly increased the hepatic protein expression levels of cluster of differentiation 36 (CD36), CPT1A and carnitine palmitoyltransferase 2 (CPT2), with the highest levels observed in the 261.90, 261.90, and 390.00 mg/kg TAs groups, respectively (*P* < 0.05). Furthermore, compared with the 0.00 mg/kg TAs group, dietary supplementation with 179.30–261.90 mg/kg TAs also significantly increased the mRNA expression of mitofusin 1 (*mfn1*) and mitofusin 2 (*mfn2*), as well as the protein expression of optic atrophy 1 (OPA1) in the liver (*P* < 0.05, Fig. 3C–E).

**Fig. 4:**
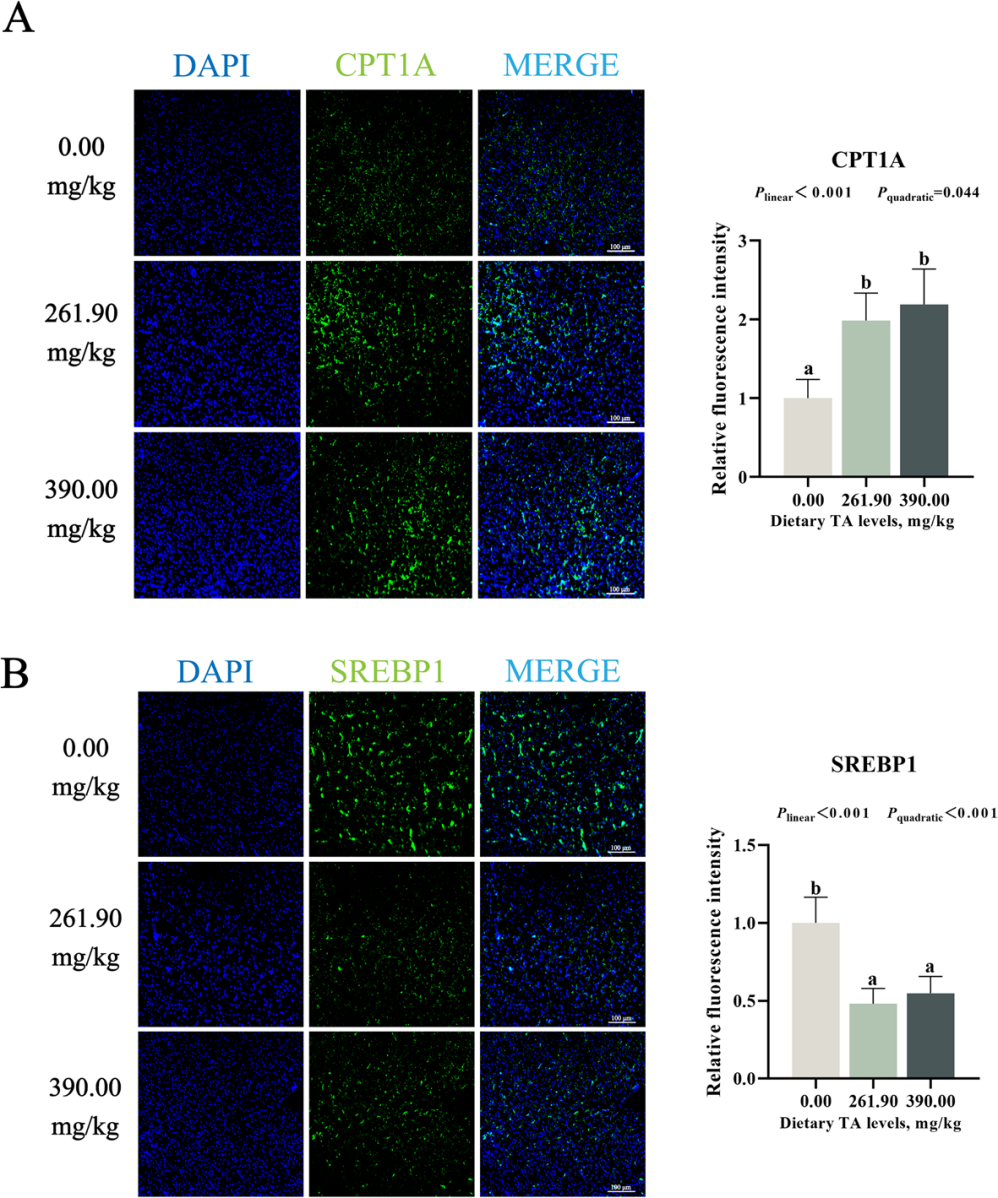
Immunofluorescence staining and quantitative analysis of liver CPT1A (**A**) and SREBP-1 (**B**). Results were expressed as mean with SD (*n* = 6). Different letters indicate significant difference (*P* < 0.05)

As shown in Fig. 3 F–H, compared with the 0.00 mg/kg TAs group, dietary supplementation with 179.30–261.90 mg/kg TAs significantly promoted the protein expression levels of hepatic PGC-1α, FXR, and PPARα, as well as the mRNA expression level of *shp*, reaching their peaks at 179.30, 261.90, 261.90, and 179.30 mg/kg TAs, respectively (*P* < 0.05). In addition, compared with the 0.00 mg/kg TAs group, dietary supplementation with 179.30–390.00 mg/kg TAs significantly suppressed SREBP1 protein expression levels, reaching its lowest level in the 312.00 mg/kg TAs group (*P* < 0.05). Moreover, immunofluorescence results showed that dietary supplementation with 261.90 and 390.00 mg/kg TAs significantly increased the immunofluorescence intensity of CPT1A and decreased the immunofluorescence intensity of SREBP1 (*P* < 0.05, Fig. 4A and B).

### Effects of TAs on FXR–SMPD3–ceramide axis in intestine

As shown in Table 5, compared with the control group, supplementation with 58.80–390.00 mg/kg TAs in the diet significantly decreased the ceramide content in the intestine, reaching the minimum in the 179.30 mg/kg TAs group (*P* < 0.05). Although no significant differences were observed among treatments (*P* > 0.05), liver ceramide content in the TA-supplemented groups was lower than in the control group. Furthermore, quadratic regression analysis revealed a significant quadratic relationship between liver ceramide content and dietary TA levels (*P* < 0.05).

**Table 5:** Effects of TAs on ceramide levels in the intestine and liver of juvenile grass carp, ng/g tissue

Item	**Dietary TA levels, mg/kg**						***P*-value**		
	**0.00**	**58.80**	**179.30**	**261.90**	**312.00**	**390.00**	**ANOVA**	**Linear**	**Quadratic**
Intestinal ceramide	1,434.88 ± 77.21^c^	1,012.26 ± 87.14^a^	985.52 ± 72.75^a^	988.96 ± 88.40^a^	1,149.26 ± 98.62^b^	1,163.62 ± 60.17^b^	< 0.001	0.002	< 0.001
Hepatic ceramide	1847.59 ± 84.99	1723.30 ± 90.18	1696.44 ± 114.63	1659.73 ± 116.23	1742.82 ± 155.47	1719.27 ± 97.22	0.120	0.117	0.032

Results were expressed as mean ± SD (*n* = 6); ^a^^–^^c^ Mean values within a row with different superscript letters indicate significant different (*P* < 0.05)

**Fig. 5:**
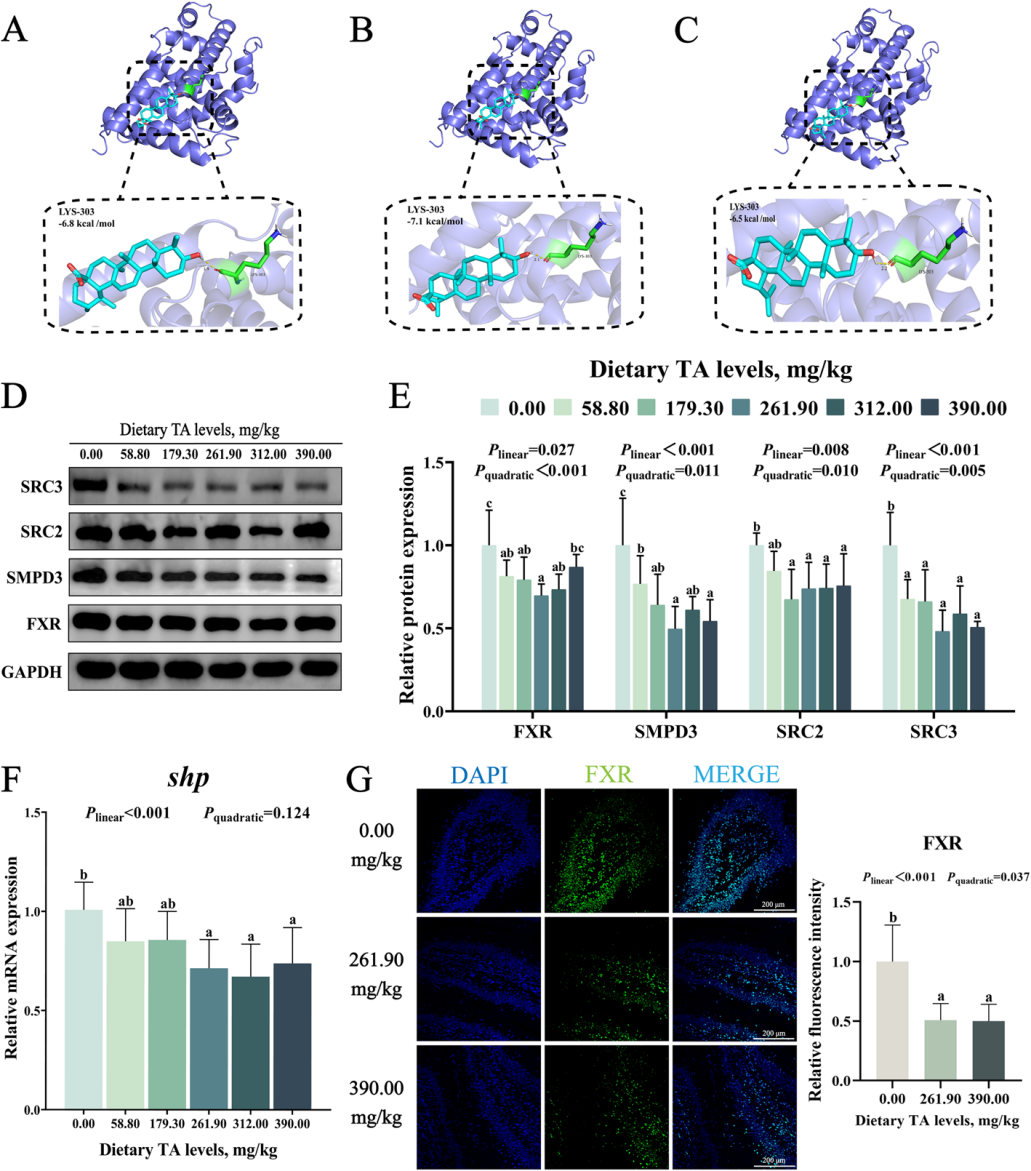
Effects of TAs on FXR–SMPD3–ceramide axis in intestine. **A–C** Molecular docking of FXR protein with ursolic acid (**A**), oleanolic acid (**B**), and betulinic acid (**C**). **D** and **E** The protein levels of FXR, SMPD3, SRC2 and SRC3 in grass carp intestine. **F** The mRNA expression of *shp* in grass carp intestine. **G** The immunofluorescence levels of FXR in grass carp intestine. Results were expressed as mean with SD (*n* = 6). Different letters indicate significant difference (*P* < 0.05)

Based on Fig. 5A–C, molecular docking revealed that ursolic acid, oleanolic acid, and betulinic acid exhibited strong binding affinities to FXR at the K303 site, with binding energies of −6.8, −7.1, and −6.5 kcal/mol, respectively. As shown in Fig. 5D and E, compared with the 0.00 mg/kg TAs group, dietary supplementation with 179.30–312.00 mg/kg TAs significantly inhibited intestinal protein expression of FXR, SMPD3, steroid receptor coactivator 2 (SRC2), and steroid receptor coactivator 3 (SRC3), reaching their lowest levels at 261.90, 261.90, 179.30, and 261.90 mg/kg TAs, respectively (*P* < 0.05). Additionally, compared with the 0.00 mg/kg TAs group, dietary supplementation with 261.90–390.00 mg/kg TAs significantly decreased intestinal *shp* mRNA expression, which reached its lowest level in the 312.00 mg/kg TAs group (*P* < 0.05, Fig. 5F). Immunofluorescence results also indicated that, compared with the 0.00 mg/kg TAs group, supplementation with 261.90 and 390.00 mg/kg TAs in the diet reduced the immunofluorescence intensity of intestinal FXR (*P* < 0.05, Fig. 5G).

### Optimal dietary TAs supplementation levels of juvenile grass carp

**Fig. 6:**
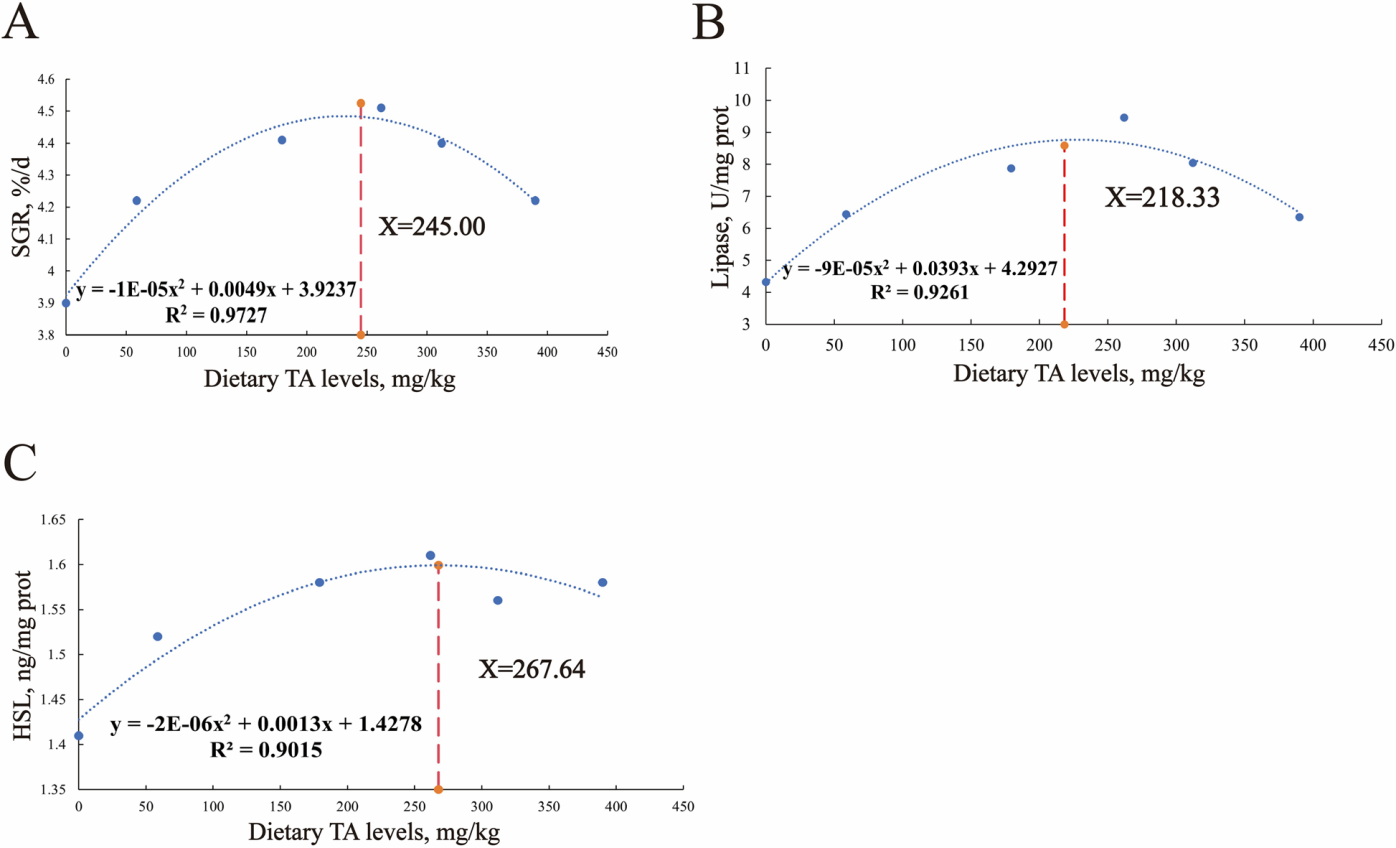
Analysis of juvenile grass carp fed diets containing graded levels of TAs for 10 weeks. **A** Specific growth rate (SGR). **B** Lipase activity. **C** Hormone-sensitive triglyceride lipase (HSL) activity

The optimal dietary TAs supplementation levels for juvenile grass carp are presented in Fig. 6. Based on SGR; the optimal dietary TAs supplementation level was determined to be 245.00 mg/kg. Additionally, based on the intestinal lipase activity and hepatic HSL activity of juvenile grass carp, the optimal TAs supplementation levels were determined to be 218.33 and 267.64 mg/kg, respectively.

## Discussion

### Appropriate levels of TAs improved growth performance and nutrient composition in fish

This study demonstrated that supplementation with 58.80–390.00 mg/kg TAs in the diet enhanced the growth performance (e.g., FBW and SGR) of juvenile grass carp, consistent with previous findings on ursolic acid in broilers [39]. Moreover, compared with the control group, dietary supplementation with 58.80–390.00 mg/kg TAs increased the FI of juvenile grass carp. Previous research has shown that rosemary extract can enhance the average daily feed intake of weaning piglets [40], suggesting that TAs may exert appetite-stimulating effects. Fish growth is dependent on nutrient accumulation [41]. Our study showed that supplementation with appropriate levels of TAs in the diet increased the CP and PRV of whole fish, while decreasing the EE. These results indicate that appropriate levels of TAs may promote more efficient utilization of lipids as an energy source, thereby reducing protein catabolism for energy and allowing greater protein allocation to tissue synthesis. However, this still requires further investigation.

The biological actions of triterpene acids are closely associated with their pharmacokinetics. P-glycoprotein, encoded by the *mdr1* gene, functions as an efflux transporter in the intestine, whereas CYP3A4 is a major metabolic enzyme in the liver [42]. A previous study has shown that ursolic acid is absorbed in the intestine mainly by passive diffusion and is partially excreted via P-glycoprotein. Subsequently, it is primarily distributed to the liver, where it is metabolized by CYP3A4 [43]. The pharmacokinetics of oleanolic acid and betulinic acid are not yet clearly defined. This study showed that dietary supplementation with 261.90–390.00 mg/kg TAs increased intestinal *mdr1* mRNA expression, and supplementation with 58.80–390.00 mg/kg TAs increased hepatic CYP3A4 protein levels in juvenile grass carp. These results suggest that TAs can be absorbed in the intestine, transported to the liver, and exert biological effects. Therefore, the following section focuses on the intestinal responses to TAs.

### Appropriate levels of TAs enhanced the digestive and absorptive capacity

The ability of the intestine to digest and absorb nutrients is closely related to fish growth and development [44]. Digestive enzymes catalyze the breakdown of proteins, lipids, and carbohydrates into absorbable small molecules [45]. Brush border enzymes are located on the surface of intestinal villi and are involved in the transmembrane transport of small molecules [46]. In this study, dietary supplementation with 179.30–312.00 mg/kg TAs increased the activities of digestive enzymes (trypsin, chymotrypsin, lipase, and amylase) and brush border enzymes (AKP, Na⁺/K⁺-ATPase, γ-GT, and CK) in the intestine, indicating that appropriate levels of TAs enhance the digestive and absorptive capacity of juvenile grass carp.

### Appropriate levels of TAs improved liver lipid metabolism through differential FXR regulation in the liver and intestine

#### Appropriate levels of TAs improved serum lipid metabolism and liver health

Reduced serum GOT and GPT levels reflect improved liver health [47]. This study demonstrated that dietary supplementation with 179.30–390.00 mg/kg TAs reduced serum GOT and GPT levels. Meanwhile, histological examination by H&E staining further showed that 261.90 and 390.00 mg/kg TAs could improve liver structure. Oil Red O staining showed that 261.90 and 390.00 mg/kg TAs could reduce the accumulation of lipid droplets in the liver. Therefore, appropriate levels of TAs improved the liver health of juvenile grass carp.

Serum TG, TC, and LDL-C reflect lipid accumulation in the body and the burden of cholesterol transport to peripheral tissues, whereas HDL-C represents the capacity for reverse cholesterol transport and is an important indicator of lipid metabolic balance [48]. Our results showed that dietary supplementation with 261.90 mg/kg TAs reduced serum TG, TC, and LDL-C levels while increasing serum HDL-C levels. Therefore, appropriate levels of TAs improved systemic lipid metabolism in juvenile grass carp.

#### Appropriate levels of TAs promoted hepatic lipid metabolism and activated hepatic FXR pathway

FAS is a key enzyme in fatty acid synthesis, while ATGL and HSL catalyze the initial and subsequent steps of triglyceride hydrolysis, thereby regulating lipolysis [49]. In this study, we found that dietary supplementation with 58.80–312.00 mg/kg TAs increased hepatic ATGL and HSL activities, while decreasing hepatic FAS activity. These results suggest that appropriate levels of dietary TAs promote hepatic lipid metabolism in juvenile grass carp.

ACACA, SCD, DGAT1A, PPARγ, and HMGCR are involved in fatty acid and cholesterol synthesis; MAGL, ACADM, and ACOX1 participate in lipid degradation and oxidation; CD36, CPT1A, and CPT2 are associated with mitochondrial transport and oxidation of fatty acids [50]. In this study, appropriate levels of TAs decreased the mRNA levels of hepatic *acaca*, *scd*, *dgat1a*, and *hmgcr*, while increasing the mRNA levels of *pparγ*, *magl*, *acadm*, *acox1*, and the protein levels of CD36, CPT1A, and CPT2. Collectively, these alterations in gene and protein expression suggest that dietary TAs regulate hepatic lipid metabolism by simultaneously inhibiting key pathways for fatty acid and cholesterol synthesis while promoting lipid degradation and mitochondrial fatty acid oxidation. Interestingly, *pparγ* levels increased after dietary supplementation with TAs, unlike other lipid synthesis–related factors. This may be attributed to the additional roles of PPARγ in lipid metabolism. Apart from promoting lipid synthesis and storage, PPARγ can also upregulate the expression of ATGL and HSL [51]. These results indicate that TAs suppress some lipid synthesis pathways while promoting lipid uptake, degradation, and oxidation.

To explore the molecular basis of these effects, we examined key transcription factors regulating lipid synthesis and degradation. SREBP-1 is a central regulator of fatty acid and triglyceride synthesis, whereas PPARα is the main driver of fatty acid oxidation [52]. This study found that supplementation with 179.30–261.90 mg/kg TAs not only downregulated SREBP-1 expression, but also upregulated PPARα expression, which is consistent with the overall impact of TAs on lipid metabolism. The effects of TAs on PPARα and SREBP-1 may be related to FXR, SHP, and PGC-1α. As an upstream nuclear receptor, FXR integrates these pathways by activating PPARα to enhance fatty acid oxidation while repressing SREBP-1 via SHP to suppress lipogenesis [53]. In addition, PGC-1α is one of the coactivators of FXR and is highly expressed in the liver [20]. This study found that dietary supplementation with 179.30–261.90 mg/kg TAs increased the protein expression of PGC-1α and FXR, and the mRNA expression of *shp* in the liver. The above results suggest that the effects of TAs on promoting lipid catabolism and inhibiting lipid synthesis are associated with activation of the hepatic FXR pathway.

Fatty acid oxidation is a key process linking lipid decomposition and utilization [54]. Mitochondria are the central sites of fatty acid oxidation and energy production, and their structural integrity is crucial for efficient hepatic lipid metabolism [55]. OPA1 is an inner mitochondrial membrane fusion protein that regulates inner membrane fusion and cristae remodeling, whereas MFN1 and MFN2, located on the outer mitochondrial membrane, mediate outer membrane fusion and maintain the continuity of the mitochondrial network [56]. Our study revealed that dietary TAs supplementation (261.90–312.00 mg/kg) increased OPA1 protein levels and upregulated *mfn1* and *mfn2* mRNA expression in the liver, suggesting that appropriate levels of TAs contribute to maintaining the structural integrity of liver mitochondria. In addition to the self-regulation of the liver, the gut–liver axis also plays a crucial role in regulating hepatic lipid metabolism. Therefore, in the next section, we discuss the impact of TAs on the gut–liver axis.

#### Appropriate levels of TAs inhibited the intestinal FXR–SMPD3–ceramide axis

Ceramide accumulation in the intestine is thought to negatively affect hepatic lipid metabolism and mitochondrial structure via the gut–liver axis [57]. This study found that dietary supplementation with 58.80–390.00 mg/kg TAs reduced ceramide levels in the intestine. Under our experimental conditions, no significant differences were observed in liver ceramide content among TAs groups. However, liver ceramide content in the TA-supplemented groups was lower than in the control group, and quadratic regression analysis revealed a significant quadratic relationship between liver ceramide content and dietary TA levels. This suggests that dietary TAs supplementation may have the potential to reduce hepatic ceramide level. In the intestine, FXR can enhance the protein level of Smpd3 (also known as neutral sphingomyelinase 2, nSMase2), which is a key enzyme for ceramide production [25]. Therefore, unlike its effects in the liver, FXR activation in the intestine inhibits hepatic lipid metabolism [24]. In this study, we found that dietary supplementation with 58.80–312.00 mg/kg TAs could reduce the expression of intestinal FXR and SMPD3. Furthermore, one study has shown that the cyclic peptide DC646 binds to K303 in the ligand-binding domain (LBD) of FXR, triggering conformational changes in the LBD and inhibiting recruitment of the coactivator SRC2 [58]. In this study, molecular docking results showed that ursolic acid, oleanolic acid, and betulinic acid all bound strongly to the FXR protein at K303. Moreover, we discovered that dietary supplementation with 179.30–390.00 mg/kg TAs decreased the protein levels of the FXR coactivators SRC2 and SRC3 in the intestine of juvenile grass carp. This may be the mechanism by which TAs inhibit FXR in the intestine. These results suggest that the beneficial effects of appropriate levels of TAs on hepatic lipid metabolism may be associated with suppression of the intestinal FXR-SMPD3-ceramide axis.

### Possible reasons for the different effects of TAs on intestinal and hepatic FXR

The above results indicate that TAs exert tissue-specific regulatory effects on FXR in juvenile grass carp. In the intestine, TAs inhibit FXR activation, while in the liver, TAs may activate FXR by promoting PGC-1α expression. This phenomenon may be attributed to the markedly higher expression of PGC-1α in energy-demanding tissues such as the liver [59]. By contrast, in the intestine, the primary function of PGC-1α is to maintain mitochondrial function and intestinal epithelial homeostasis [60]. Moreover, inhibition of intestinal FXR has been reported to reduce intestinal secretion of FGF15/19, which normally travels via the circulation to the liver to suppress CYP7A1 expression, thereby indirectly enhancing hepatic FXR activity [61]. The precise mechanism underlying the tissue-specific regulation of FXR by TAs remains to be further elucidated.

### Optimal dietary TAs supplementation levels of juvenile grass carp

Based on SGR, intestinal lipase activity, and hepatic HSL activity of juvenile grass carp, the optimal dietary TAs supplementation levels were estimated to be 245.00, 218.33, and 267.64 mg/kg, respectively. The optimal TAs supplementation level based on intestinal lipase activity was lower than that based on SGR, likely because dietary TAs reach the intestine first after ingestion. In contrast, the optimal level determined by hepatic HSL activity was slightly higher than those derived from SGR and lipase activity, possibly reflecting a higher TAs requirement for regulating hepatic lipid metabolism.

## Conclusion

**Fig. 7:**
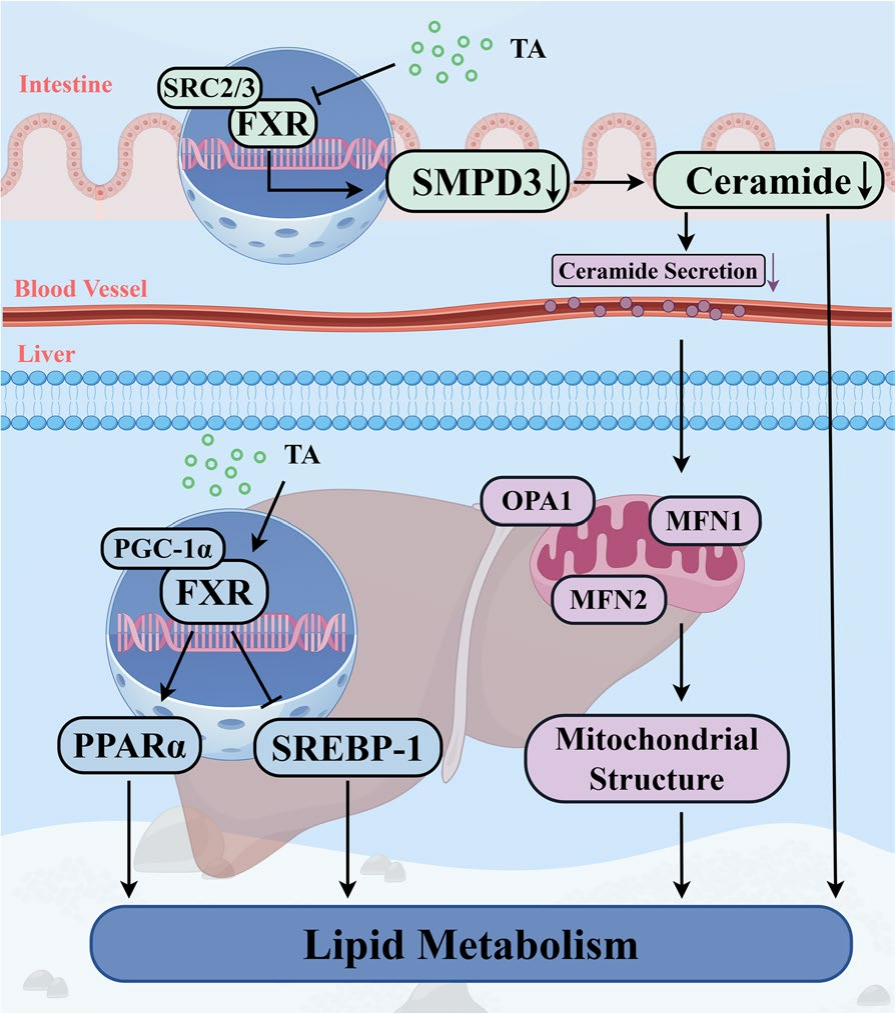
Potential mechanism of TAs improving liver lipid metabolism of juvenile grass carp

In summary, this study demonstrated for the first time that dietary supplementation with appropriate levels of TAs can enhance growth performance, improve digestive and absorptive capacity, and modulate lipid metabolism in juvenile grass carp. The effects of TAs on lipid metabolism in juvenile grass carp may be related to the following two mechanisms: (1) supplementation with appropriate levels of TAs can activate the hepatic FXR pathway and promote hepatic lipid utilization; and (2) supplementation with appropriate levels of TAs can inhibit the intestinal FXR–SMPD3–ceramide axis, which may reduce hepatic lipid deposition (Fig. 7). Finally, based on SGR, intestinal lipase activity, and hepatic HSL activity, the optimal dietary TAs supplementation levels for juvenile grass carp were determined to be 245.00, 218.33 and 267.64 mg/kg, respectively.

## Supplementary Information

Additional file 1: Full uncropped blots images.

Additional file 2: Table S1. Real-time qPCR primer sequences. Table S2. The primary antibody information of western blot analysis. Table S3. The primary antibody information of immunofluorescence staining.

## Data Availability

The datasets are included in this article and available from the corresponding author on reasonable request.

## Abbreviations

ACADM: Medium-chain acyl-CoA dehydrogenase
ACACA: Acetyl-CoA carboxylase alpha
ACOX1: Acyl-CoA oxidase 1
AKP: Alkaline phosphatase
ARV: Ash retention value
ATGL: Adipose triglyceride lipase
CD36: Cluster of differentiation 36
CK: Creatine kinase
CP: Crude protein
CPT1A: Carnitine palmitoyltransferase 1A
CPT2: Carnitine palmitoyltransferase 2
CYP3A4: Cytochrome P450 3A4
DGAT: Diacylglycerol O-acyltransferase
EE: Ether extract
FCR: Feed conversion ratio
FE: Feed efficiency
FXR: Farnesoid X receptor
FBW: Final body weight
FI: Feed intake
FAS: Fatty acid synthase
γ-GT: Gamma-glutamyl transferase
GPT: Glutamate pyruvate transaminase
GOT: Glutamate oxaloacetate transaminase
HDL-C: High-density lipoprotein cholesterol
HMGCR: 3-Hydroxy-3-methylglutaryl-CoA reductase
HSI: Hepatosomatic index
HSL: Hormone-sensitive lipase
IBW: Initial body weight
ISI: Intestinosomatic index
LDL-C: Low-density lipoprotein cholesterol
LRV: Lipid retention value
MAGL: Monoacylglycerol lipase
MDR1: Multidrug resistance 1
MFN1: Mitofusin 1
MFN2: Mitofusin 2
Na⁺/K⁺-ATPase: Sodium-potassium adenosine triphosphatase
OPA1: Optic atrophy 1
PER: Protein efficiency ratio
PGC-1α: Peroxisome proliferator-activated receptor gamma coactivator 1 alpha
PPARα: Peroxisome proliferator-activated receptor alpha
PPARγ: Peroxisome proliferator-activated receptor gamma
PRV: Protein retention value
SCD: Stearoyl-CoA desaturase
SGR: Specific growth rate
SHP: Small heterodimer partner
SRC2: Steroid receptor coactivator 2
SRC3: Steroid receptor coactivator 3
SMPD3: Sphingomyelin phosphodiesterase 3
SREBP-1: Sterol regulatory element-binding protein 1
TAs: Rosemary-derived triterpene acids
TC: Total cholesterol
TG: Triglyceride
