# Supplementary material for: Rosemary-derived triterpene acids improve growth and lipid metabolism in juvenile grass carp (Ctenopharyngodon idella) through the gut–liver axis by tissue-specifically regulating the farnesoid X receptor
Source: J Anim Sci Biotechnol. 2026 Mar 12;17:46. doi: 10.1186/s40104-025-01351-1 (PMC12980912; doi:10.1186/s40104-025-01351-1)

CYP3A4 in liver

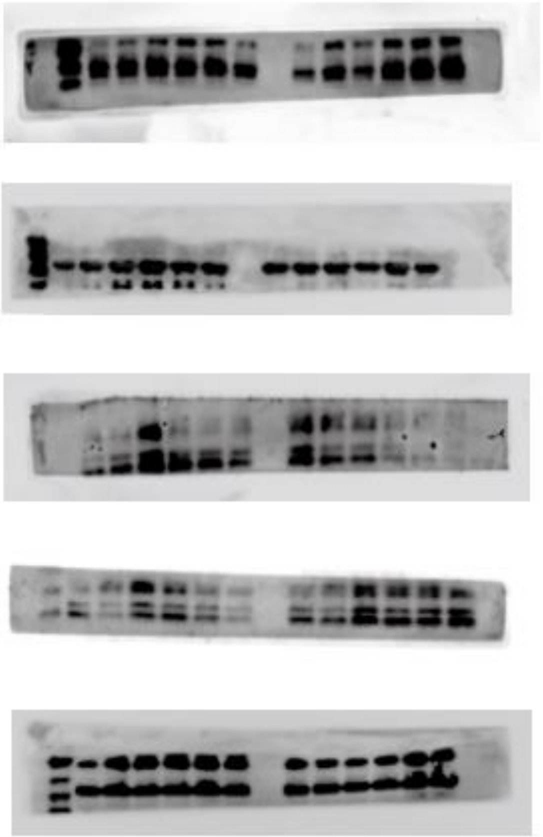

GAPDH in liver

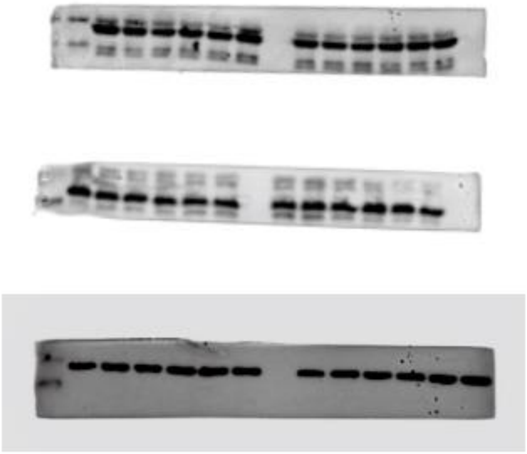

CPT1A in liver

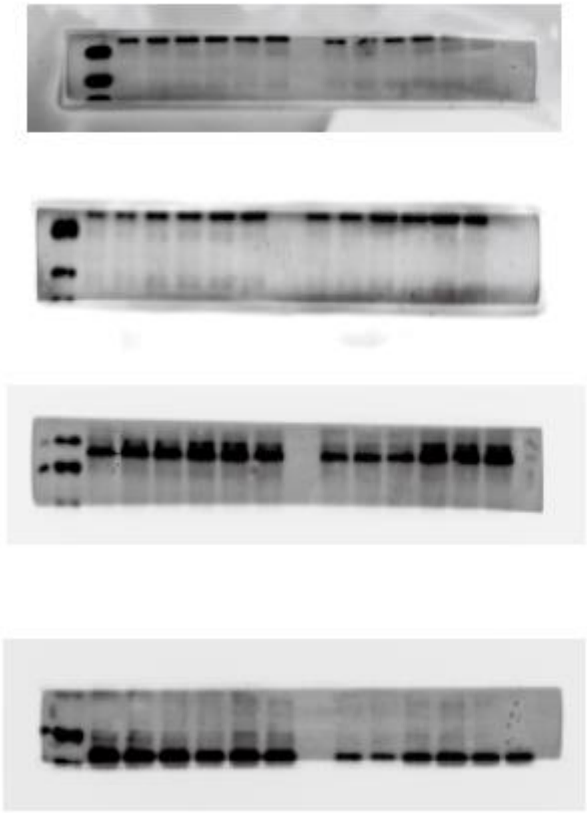

CD36 in liver

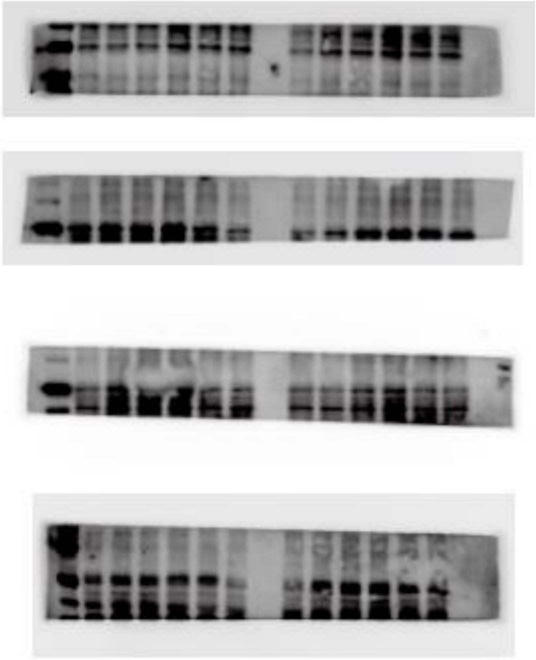

CPT2 in liver

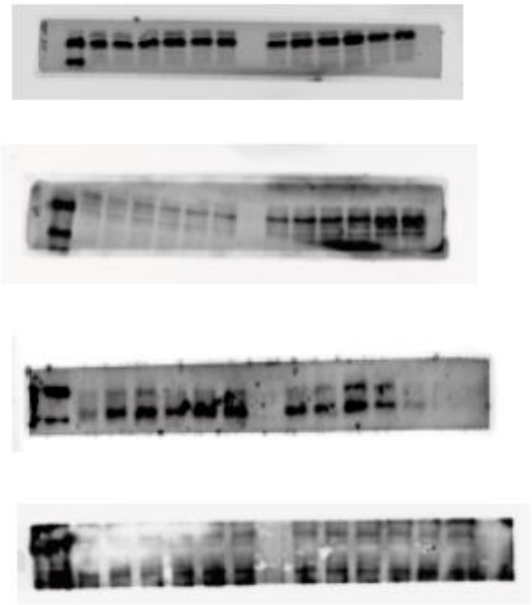

OPA1 in liver

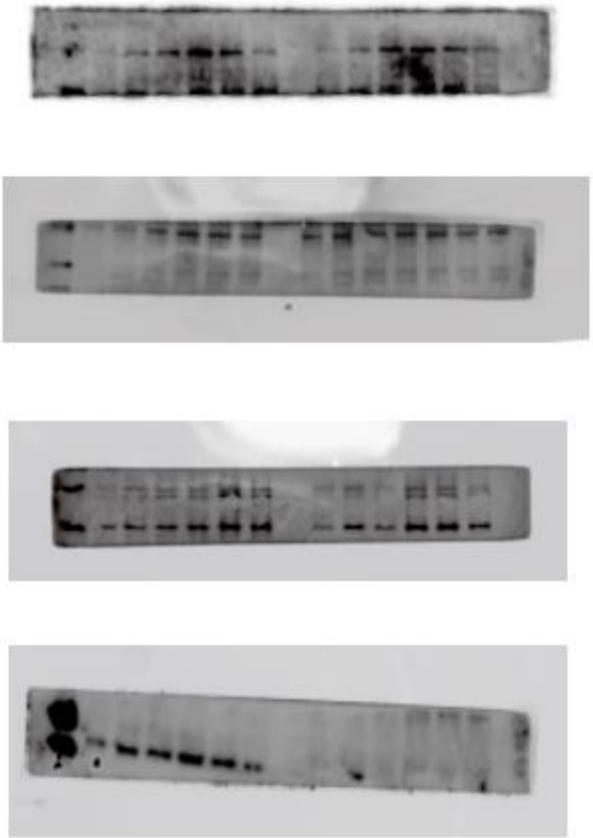

PGC1α in liver

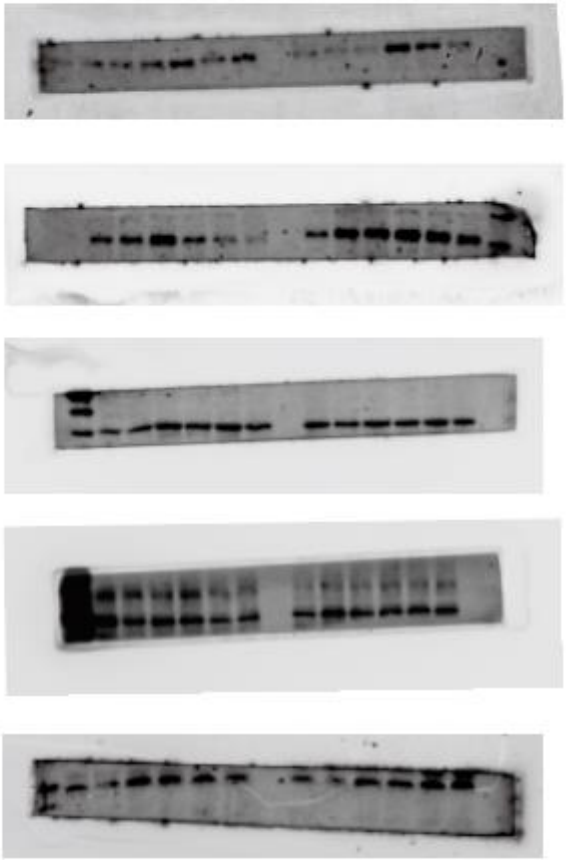

FXR in liver

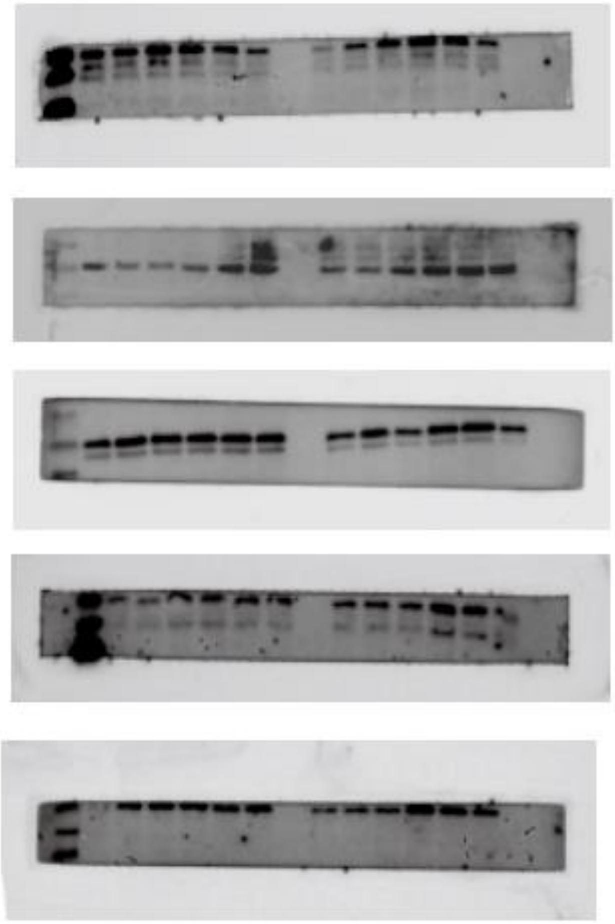

PPARα in liver

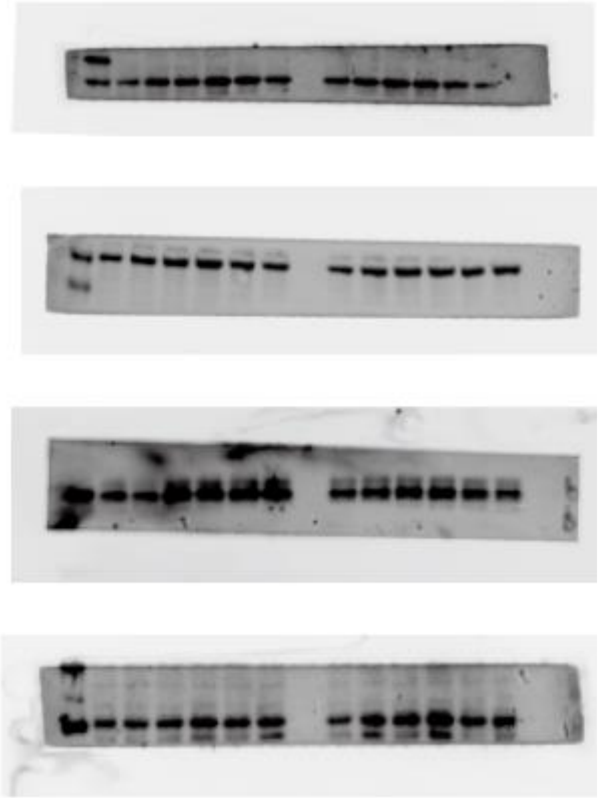

SREBP1 in liver

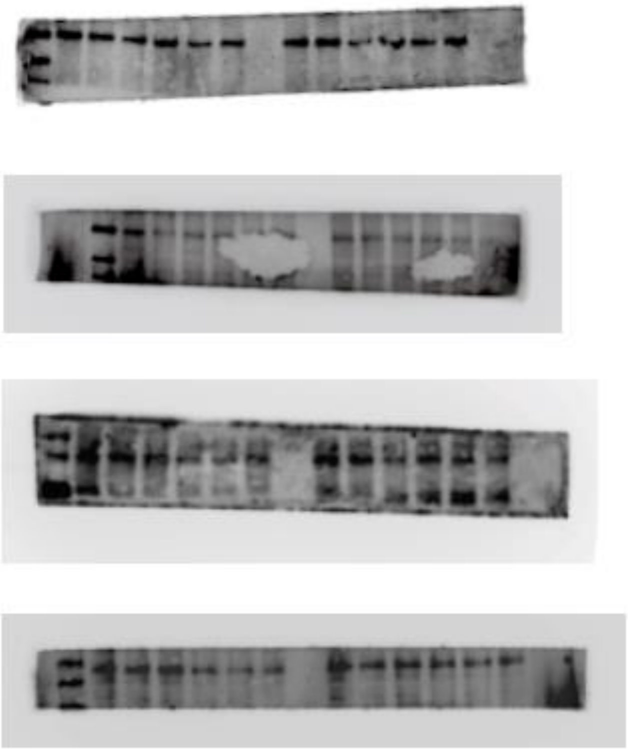

GAPDH in Intestine

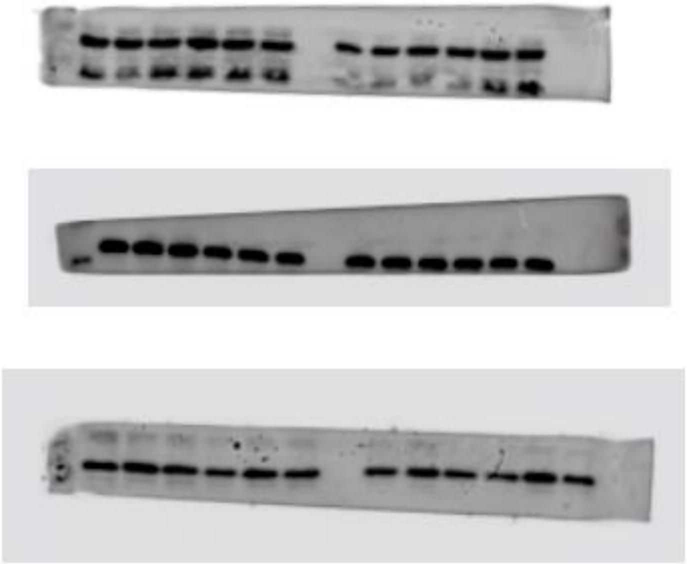

FXR in intestine

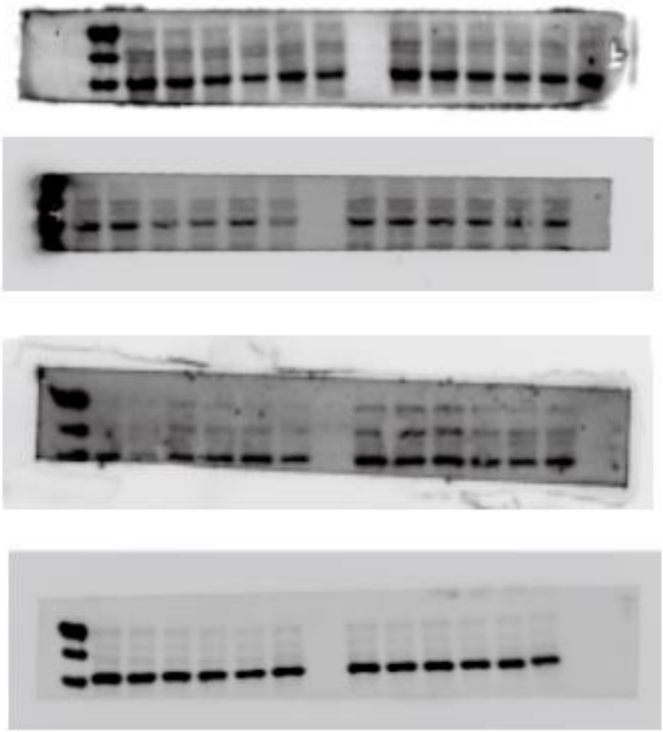

SMPD3 in intestine

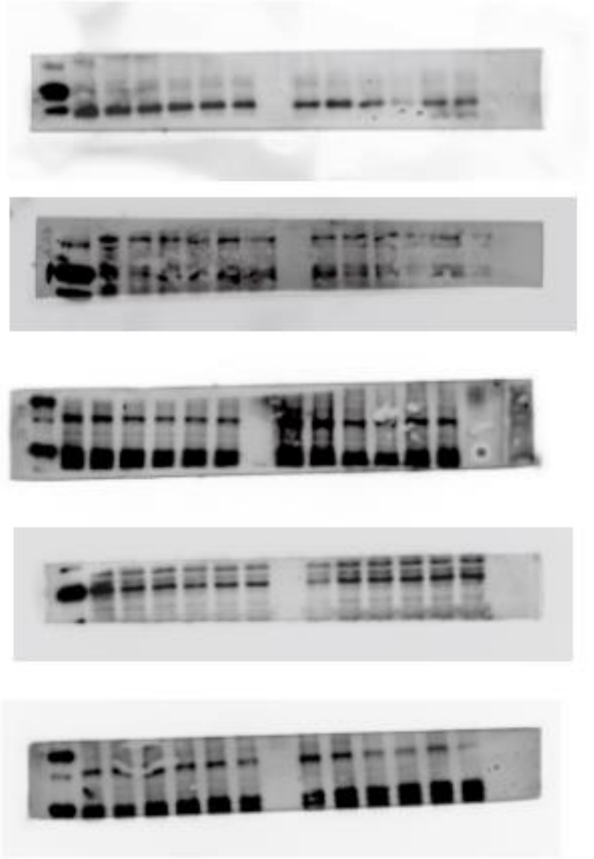

SRC2 in intestine

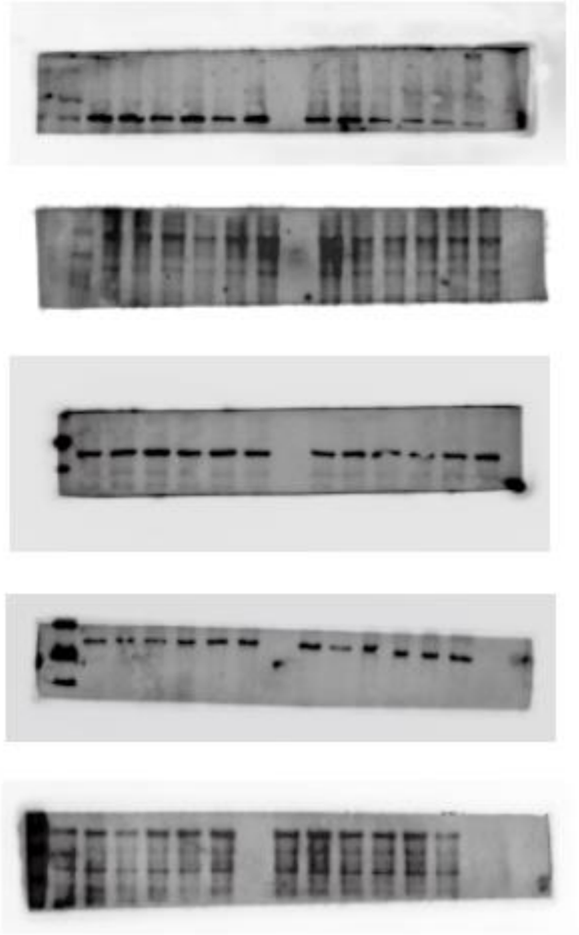

SRC3 in intestine

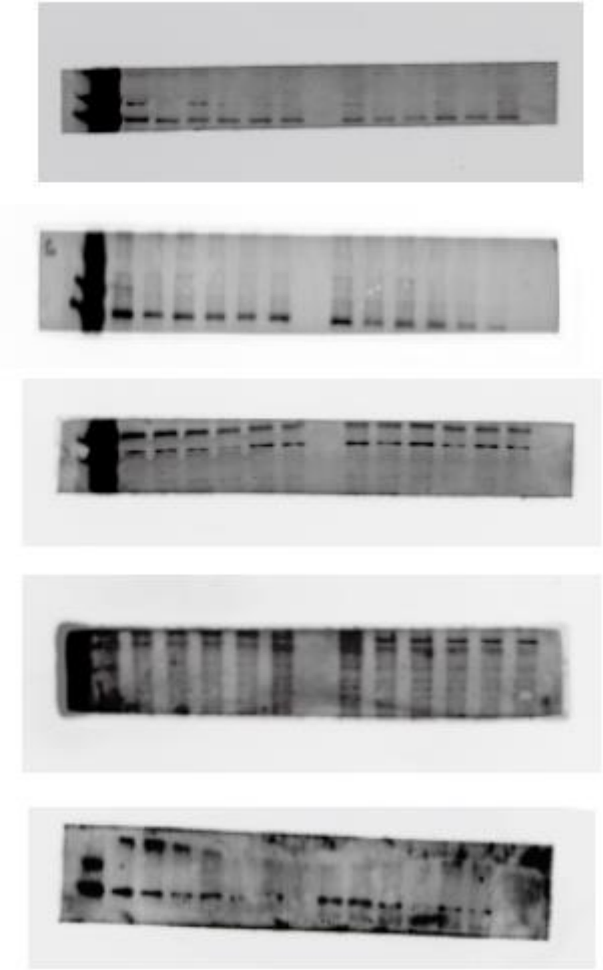

Supplement: Supplementary file 1 — Additional file 1: Full uncropped blots images. [file 40104_2025_1351_MOESM1_ESM.pdf]
