# Supplementary material for: Rosemary-derived triterpene acids improve growth and lipid metabolism in juvenile grass carp (Ctenopharyngodon idella) through the gut–liver axis by tissue-specifically regulating the farnesoid X receptor
Source: J Anim Sci Biotechnol. 2026 Mar 12;17:46. doi: 10.1186/s40104-025-01351-1 (PMC12980912; doi:10.1186/s40104-025-01351-1)
Supplement: Supplementary file 2 — Additional file 2: Table S1. Real-time qPCR primer sequences. Table S2. The primary antibody information of western blot analysis. Table S3. The primary antibody information of immunofluorescence staining. [file 40104_2025_1351_MOESM2_ESM.docx]

Table S1 The primer sequences and accession numbers of genes selected for analysis by real-time PCR.

| **Target gene** | **Primer sequence forward (5′ to 3′)** | **Primer sequence reverse (5′ to 3′)** | **Product size(bp)** | **Annealing temperature (℃)** | **PCR efficiency (%)** | **R^2^** | **r** | **Accession number** |
| --- | --- | --- | --- | --- | --- | --- | --- | --- |
| *dgat* | CACCTTCCAAGTACCTTCTG | AGATCCCACTCGCCTATT | 292 | 60 | 97.78% | 0.997 | 0.998 | XM_051873340.1 |
| *scd* | GCCTTCCAGAATGACATCTAC | GCCGATGTGAGCAAAGAA | 117 | 60 | 104.35% | 0.999 | 0.999 | XM_051914897.1 |
| *acaca* | TGGAGGTGGCCTTCAACAATACCA | AAGGGTCCATGATGACAGTTGGGA | 87 | 60 | 103.09% | 0.998 | 0.999 | XM_051892870.1 |
| *magl* | TGGGAGGCATTTCGGTTAGG | ACAAACGGAGCCATAGGTGA | 174 | 60 | 94.64% | 0.991 | 0.996 | XM_051874220.1 |
| *acox1* | GTGCAGAAAAGACACGCAGG | GGGCTCTTGATACACACCCT | 221 | 60 | 95.98% | 0.994 | 0.997 | XM_051895864.1 |
| *pparγ* | CGAGTTCTCCGTCAAGTTCAA | CGCAGGTCCGTCATCTTCT | 225 | 60 | 97.64% | 0.992 | 0.996 | XM_051913344.1 |
| *hmgcr* | ATACCTCTGCACTCCACTAA | ACGAATAAAGATGGAGATGGAC | 176 | 60 | 99.10% | 0.999 | 0.999 | XM_051892838.1 |
| *acadm* | TGAACATGGGCCAGAGATGC | AGCGTACTTTGTGGCCTCTT | 197 | 60 | 96.09% | 0.991 | 0.995 | XM_051883575.1 |
| *shp* | GGCCTGAAGGCATCTCTGTT | GCAGTGAGGAGGATACGAGC | 122 | 60 | 101.40% | 0.997 | 0.998 | [1] |
| *mdr1* | CACCTGGACGTTACCAAAGAAGATATA | TCACCAACCAGCGTCTCATATTT | 127 | 60 | 96.39% | 0.995 | 0.998 | [2] |
| *mfn1* | AGTTTGGATGCTGGCTGTCT | GCTTTGAGAGGCGTTCGTTG | 238 | 60 | 95.35% | 0.997 | 0.998 | [3] |
| *mfn2* | AAAGTGGCAGGGATTGGGG | CGTAGGAAGCAGTTGGTGGT | 155 | 60 | 98.58% | 0.993 | 0.997 | [3] |
| *β-Actin* | GGCTGTGCTGTCCCTGTA | GGGCATAACCCTCGTAGAT | 101 | 60 | 99.61% | 0.992 | 0.996 | M25013 |
| *gapdh* | GTTACAAGGGAGAAGTTCACCAT | CCGGTAGACTCGACTACATACAG | 136 | 60 | 99.76% | 0.990 | 0.995 | [4] |

R², coefficient of determination. r, Pearson’s correlation coefficient.

Table S2 The primary antibody information of western blot analysis.

| Antibody name | Company | Article number | Dilution rate |
| --- | --- | --- | --- |
| FXR | HUABIO | ER1914-12 | 1:1000 |
| SMPD3 | ABclonal | A23417 | 1:2000 |
| SRC2 | HUABIO | ER63637 | 1:1000 |
| SRC3 | HUABIO | ER65603 | 1:1000 |
| OPA1 | HUABIO | HA722673 | 1:4000 |
| SREBP1 | HUABIO | HA722160 | 1:2000 |
| PGC1α | ABclonal | A12348 | 1:1000 |
| CPT1A | HUABIO | HA723261 | 1:2,000 |
| CPT2 | HUABIO | ET1611-64 | 1:1,000 |
| CD36 | ABclonal | A5792 | 1:2000 |
| PPARα | HUABIO | EM1707-71 | 1:1,000 |
| CYP3A4 | ABclonal | A2544 | 1:1,000 |
| GAPDH | Servicebio | GB15004-100 | 1:3000 |

Table S3 The primary antibody information of Immunofluorescence analysis.

| Antibody name | Company | Article number | Dilution rate |
| --- | --- | --- | --- |
| FXR | HUABIO | ER1914-12 | 1:200 |
| SREBP1 | HUABIO | HA722160 | 1:200 |
| CPT1A | HUABIO | HA723261 | 1:200 |

References

1. Wang B-B. Combined effects of berberine with Pennisetum giganteum powder or Clostridium butyricum supplementation on grass carp [Doctoral dissertation]. Beijing, China: Chinese Academy of Agricultural Sciences. 2024. <https://doi.org/10.27630/d.cnki.gznky.2024.000902>.

2. Hu K, Li H-R, Ou R-J, Li C-Z, Yang X-L. Tissue accumulation and toxicity of isothiazolinone in *Ctenopharyngodon idellus* (grass carp): Association with P-glycoprotein expression and location within tissues. Environ Toxicol Pharmacol. 2014;37:529–35. <https://doi.org/10.1016/j.etap.2013.12.017>.

3. Zeng S, Zhou X, Feng L, Wu P, Liu Y, Ma Y, et al. Glutathione mitigates hypoxia stress-induced liver injury in juvenile grass carp (*Ctenopharyngodon idellus*) by alleviating mitochondrial dysfunction and autophagy. Aquaculture. 2025;604:742489. <https://doi.org/10.1016/j.aquaculture.2025.742489>.

4. Dong Jie. Studies on the stability of four candidate internal reference genes and molecular cloning and expression of two immunization-associated genes in grass carp, *Ctenopharyngodon idellu*s. [Doctoral dissertation]. Yanglin, China: Northwest A&F University. 2010.
